# Arachidonate 15-lipoxygenase-mediated production of Resolvin D5_n-3 DPA_ abrogates pancreatic stellate cell-induced cancer cell invasion

**DOI:** 10.3389/fimmu.2023.1248547

**Published:** 2023-11-16

**Authors:** Gabriel A. Aguirre, Michelle R. Goulart, Barts Pancreas Tissue Bank, Jesmond Dalli, Hemant M. Kocher

**Affiliations:** ^1^Centre for Tumour Biology, Barts Cancer Institute, London, United Kingdom; ^2^William Harvey Research Institute, Barts and The London School of Medicine and Dentistry, John Vane Science Centre, Queen Mary University of London, London, United Kingdom

**Keywords:** pancreatic ductal adenocarcinoma, specialized pro-resolving mediator, lipid mediator, cancer-associated fibroblast, all-*trans* retinoic acid, ALOX15, CAF subtypes

## Abstract

Activation of pancreatic stellate cells (PSCs) to cancer-associated fibroblasts (CAFs) is responsible for the extensive desmoplastic reaction observed in PDAC stroma: a key driver of pancreatic ductal adenocarcinoma (PDAC) chemoresistance leading to poor prognosis. Specialized pro-resolving mediators (SPMs) are prime modulators of inflammation and its resolution, traditionally thought to be produced by immune cells. Using liquid chromatography–tandem mass spectrometry (LC-MS/MS)-based lipid mediator profiling PSCs as well as primary human CAFs express enzymes and receptors to produce and respond to SPMs. Human PSC/CAF SPM secretion profile can be modulated by rendering these cells activated [transforming growth factor beta (TGF-β)] or quiescent [all-*trans* retinoic acid (ATRA)]. ATRA-induced nuclear translocation of arachidonate-15-lipoxygenase (ALOX15) was linked to increased production of n-3 docosapentaenoic acid-derived Resolvin D5 (RvD5_n-3 DPA_), among other SPMs. Inhibition of RvD5_n-3 DPA_ formation increases cancer cell invasion, whereas addback of this molecule reduced activated PSC-mediated cancer cell invasion. We also observed that circulating concentrations of RvD5_n-3 DPA_ levels were decreased in peripheral blood of metastatic PDAC patients when compared with those measured in plasma of non-metastatic PDAC patients. Together, these findings indicate that RvD5_n-3 DPA_ may regulate cancer–stroma cross-talk and invasion.

## 1 Introduction

The poor prognosis of patients with pancreatic ductal adenocarcinoma (PDAC) (1) is believed to be due to delayed diagnosis and the histologically well-recognized complex, poorly vascularized extensive desmoplastic reaction providing a fortress for cancer cells to be shielded from therapeutic approaches. This characteristic desmoplastic stroma, made up of an excess of extracellular matrix (ECM) and different cell types, is believed to be orchestrated by the activation of the normally quiescent, homeostatic pancreatic stellate cells (PSCs) to cancer-associated fibroblasts (CAFs) (2), which, in turn, cross-talk with not only cancer cells but also a plethora of other non-transformed cells such as immune cells including M2 macrophages and anti-tumoral CD8^+^ T cells (3, 4). Recently, stromal reprogramming (of PSCs) has met success in early-phase clinical trials (5), opening an underexplored avenue to reverse the activated stroma to a homeostatic, physiological state along with chemotherapy to target pancreatic cancer cells. The precise mechanism(s) of the multitude of cross-talk has been restricted to protein signaling molecules such as Wnt-β-catenin (6), CXCL12 (4), RARβ (7), PTX3 (8), CCL21, and CXCL13 (9) with scant attention being paid to lipid-derived signaling molecules.

PSCs, which are resident fibroblasts of the pancreas, are the major source of CAFs in PDAC. In their normal physiological quiescent state, PSCs store retinyl ester-containing lipid droplets (mainly vitamin A) (10). Upon activation, PSCs lose this storage function, express α-smooth muscle actin (αSMA), and secrete ECM proteins, remodeling enzymes, and growth factors, transdifferentiating into myofibroblasts (10). Recently, CAF subtypes in PDAC have been recognized with coexisting pro- and anti-tumoral subpopulations (11–18). Such heterogeneity may account for the paradoxical effects observed in stromal depletion studies (5, 19–25). Exploiting diversity in CAF phenotypes may shape the future of co-adjuvant anti-stroma therapies.

In recent reports, four CAF subtypes (A to D), with specific molecular and functional features, were described using primary cultures from resected PDAC-derived human CAFs. Based on transcriptomic and immunohistochemistry (IHC) analysis, periostin (POSTN), myosin-11 (MYH11), and podoplanin (PDPN) were identified as markers of subtypes A, B, and C, respectively. POSTN-positive CAFs (subtype A) were the most frequently present and were associated with poor prognosis in patients with resected PDAC (11, 12). Moreover, CAF phenotyping permitted patient prognostic stratification and may also be explored as predictive markers of response to immune therapy (25). Also, CAF subtypes, by their association with basal-like/classical subtypes, may modulate tumor sensitivity to chemotherapy (11, 12, 25). In this sense, reprogramming pro-tumorigenic CAF populations (e.g., POSTN^+^—A or D subtypes) may emerge as a new co-adjuvant therapeutic strategy in PDAC.

Resolution of inflammation, classically believed to be achieved by passive withdrawal of the pro-inflammatory mediators after insult cessation, is increasingly recognized as an active process (26). Systematic studies evaluating the cellular and biochemical processes occurring during this phase of the inflammatory response uncovered a role for lipid mediator class switching, from the classical eicosanoids to the specialized pro-resolving mediators (SPMs) that carry anti-inflammatory and pro-resolving actions. SPMs include lipoxins (LX), resolvins (Rv), protectins (PD), and maresins (MaR) (27).

SPMs are generated by the sequential action of oxygenating enzymes (lipoxygenases and cyclooxygenases) on arachidonic acid (AA), eicosapentaenoic acid (EPA), docosahexaenoic acid (DHA), and n-3 docosapentaenoic acid (n-3 DPA) (26). To carry out their job in mammalian cellular systems, oxygenating enzymes need cleaved polyenoic fatty acids from ester lipids. This is done by the catalytic activity of ester lipid hydrolyzing enzymes (e.g., ALOX5 preferentially interacts with phospholipase A2) (28). The hydroperoxy fatty acids formed by ALOXs are subsequently converted to a large array of bioactive lipid mediators, which include leukotrienes, hepoxilins, eoxins, and SPMs, among others (28, 29). In order to produce SPMs, COX2, ALOX5, ALOX12, ALOX15, and ALOX15B are needed, along with other hydrolases and cytochromes (26, 29–31). Differential expression of various ALOXs/COXs, their binding proteins, and their subcellular localization may enhance the production of specific precursors and products (29–31). Once lipid mediators are produced, they can exert their biological actions through several mechanisms. SPMs are known to activate G-protein-coupled receptors (GPCRs) differentially, but still many SPMs have no recognized cognate or specific receptor (32, 33).

While SPMs are extensively studied in the context of acute and chronic inflammation, recent data suggest that specific classes of resolvins may, in fact, modulate tumor and immune cell behavior. For example, in a transplantation murine model, chemotherapy-induced tumor cell debris can enhance tumor growth *in vivo* by modulating macrophage activation (34, 35). Resolvins (RvD1, RvD2, or RvE1) increase debris phagocytosis by macrophages and counter-regulate macrophage release of cytokines/chemokines, resulting in reduced tumor growth, indicating an indirect effect on tumor growth (35). In fact, chemotherapy and surgery in cancer can induce an inflammatory/immunosuppressive injury that provokes dormancy escape leading to tumor recurrence (34). Pre-operative (but not post-operative) administration of ketorolac (preferentially inhibits COX-1) and resolvins eliminated micro-metastases in different tumor resection models, resulting in long-term survival. The study found that ketorolac and SPMs triggered antitumor T-cell immunity that synergized with immune checkpoint blockade (34). Moreover, LXA_4_, LXB_4_, RvD1, and RvD2 were all found elevated in PDAC patients compared to healthy individuals with no correlation to TNM staging but good sensitivity and specificity for detecting PDAC (36). These findings support the potential role of SPM in PDAC and the importance of evaluating their potential utility in understanding both disease processes and novel therapeutic leads.

Lipid mediators, including SPMs, have scarcely been explored in PDAC stromal context. This is surprising given the critical role of lipid signaling molecules in cross-talk with immune cells, and the central role of disordered lipid absorption and metabolism in patients with PDAC. To address this knowledge gap, we explored the role of SPMs in PSC activation to evaluate their role in limiting disease progression and their potential utility as diagnostic markers. In the present report, we identify differences in these populations that could explain how they regulate their phenotype or cross-talk through lipid mediators that could potentially be targeted or used as biomarkers.

## 2 Materials and methods

### 2.1 Cell culture

Healthy human, telomerase reverse transcriptase (hTERT) immortalized, pancreas-derived stellate cells (PS1) (37) and human primary CAFs (12) were grown as previously described. PS1s and CAFs were rendered quiescent by daily treatment with 1 µM all-*trans* retinoic acid (ATRA; R2625, Sigma-Aldrich) under subdued light conditions daily for 7 days or with contemporary vehicle (ethanol 0.01% v/v) controls (6, 7). Two pancreatic cancer cell lines, AsPC-1 (CRL-1682, ATCC) and MIAPaCa-2 (CRL-1420, ATCC), based on their representation of PDAC diversity were employed (38). All cell lines were STR profiled and tested negative for mycoplasma.

### 2.2 Organotypic culture model

Pancreatic cancer organotypic cultures were constructed as previously described (39) and quantified (**Supplementary Methods**). Gels were treated from the media underneath and replaced with fresh media daily for 10 days with either ATRA 1 µM, Zileuton 10 µM (Cayman, USA), PD146176 5 nM (Cayman, USA), RvD5_n-3 DPA_ 1/10 nM (Cayman, USA), vehicle (ethanol 0.01%), or a combination of these treatments.

### 2.3 Hanging droplet spheroid model

Prior to spheroid generation, PS1 cells were pre-treated for 7 days. 3D spheroid cancer cell/PS1 co-cultures were generated using a hanging drop spheroid model developed by Ed Carter/Richard Grose (BCI-QMUL), based on previous work (40) (**Supplementary Methods**).

### 2.4 Lipid mediator profiling by coupled liquid chromatography with tandem mass spectrometry

PS1 or CAFs (one patient per subtype) cells treated with either ATRA 1 µM for 7 days daily, TGF-β 5 ng/mL (100-21, Preprotech; only PS1) for 3 days daily, or vehicle (ethanol 0.01%v/v) were analyzed by LC-MS/MS using an LC-20AD HPLC and a SIL-20AC autoinjector (Shimadzu Corp) paired with a QTrap 6500+ (AB Sciex) MS/MS, as described previously (41). Details are provided in **Supplementary Methods**. LC-MS/MS data can be found in The BioStudies database (https://www.ebi.ac.uk/biostudies/studies/ (42)) under the following accession numbers: S-BSST856 (PS1 cells), S-BSST859 (CAF cells), and S-BSST860 (PDAC patient plasma).

### 2.5 Western blot, RT-qPCR, MTS assays, and immunocytochemistry

Previously described (7, 8, 40), well-established techniques were used (**Supplementary Methods**).

### 2.6 Patient samples

Plasma from 20 PDAC patients and 22 age- and gender-matched healthy controls was collected from the Barts Pancreas Tissue Bank (REC approval: 18/SC/0630, projects 2019/12/QM/HK/P/Blood, 2020/06/QM/HK/E/Blood). PDAC patients were treatment naïve before surgery and had no diabetes or inflammatory conditions.

### 2.7 ALOX15 shRNA knockdown cell line generation

Four different ALOX15 human short hairpin RNA (shRNA) green fluorescent protein (GFP) lentiviral plasmids (TL314822, Origene) were constructed in pGFP-C-shLentivectors, with cloning in One Shot Stb13 *E. coli* (C7373-03, ThermoFisher) using chloramphenicol selection and puromycin as mammalian cell selection antibiotics. Top 10% GFP-positive cells after shRNA GFP-lentiviral vector transfection were selected using flow cytometry-assisted cell sorting (FACS).

### 2.8 Statistics

Statistical significance relied on Welch’s and Student’s *t*-test if the data satisfied the assumptions of normality and homogeneity of variance tested by the Kolmogorov–Smirnov, or a relevant test (GraphPad Prism Version 8.0). Data that failed the assumption of heteroscedasticity were analyzed using the Mann–Whitney *U*-test. For organotypic data analysis, a one-way ANOVA test was used with appropriate post-hoc corrections. PLSDA, PCA, and VIP scores were determined using Metaboanalyst (43), and pathways analysis was conducted using Cytoscape (44) using normalized (mean-centered and divided by the standard deviation of each mediator) mean concentrations for mini-heatmaps or concentration fold change for patient data. For all analyses, *p* < 0.05 was considered statistically significant. False discovery rate (FDR) correction was applied for lipid mediator analysis.

## 3 Results

### 3.1 PSCs produce specialized pro-resolving mediators and their profiles change reflecting PSC activation status

**Figure 1:**
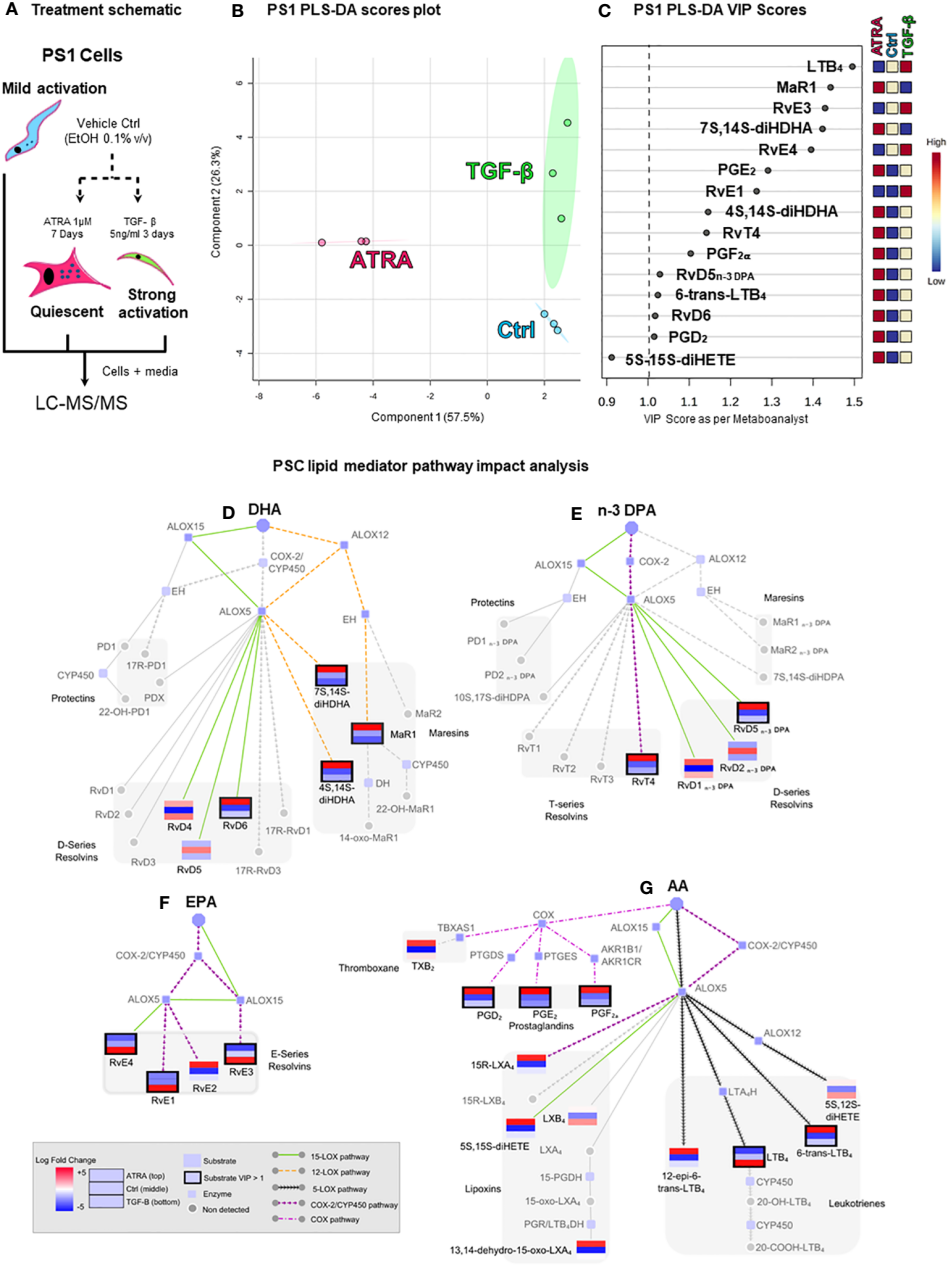
Pancreatic stellate cells’ (PSCs’) lipid mediator profiling dependent on activation status. **(A)** Schematic depiction of the experiment: cultured PSCs (PS1) were treated daily with ATRA 1 µM (quiescent) for 7 days, with TGF-β 5 ng/mL (strongly activated) for 3 days, or with vehicle control (Ctrl, ethanol 0.01%, mildly activated by plastic culturing). Cells were then lysed with 100% methanol containing deuterated internal standards along with the culture media and stored at −80°C to allow for protein precipitation and subsequent lipid mediator extraction and identification using LC-MS/MS-based lipid mediator profiling. **(B)** Partial least squares discriminant analysis (PLS-DA, supervised) scores plot depicting the three biological replicates from Ctrl-, ATRA-, and TGF-β-treated PS1 cells. **(C)** PLS-DA variable in projection (VIP) score plot highlighting all significant mediators (VIP > 1) contributing to the separation of the clusters of the three conditions (Ctrl, ATRA, and TGF-β). **(D–G)** Pathway analysis highlighting the mediators that were found differentially regulated in PLS-DA analysis and their synthetic pathways. Each mediator is shown as a mini-heatmap depicting their relative concentration among the different conditions in PS1 cells: ATRA (top rectangle), Ctrl (middle), and TGF-β (bottom). Same pattern connecting lines indicate a synthetic pathway. Grayed-out analytes were not detected. Dark border highlight indicates a VIP > 1. Figure is split according to lipid mediator metabolomes: **(D)** docosahexaenoic acid (DHA), € n-3-docosapentaenoic acid (n-3 DPA), **(F)** eicosapentaenoic acid (EPA), or **(G)** arachidonic acid (AA). n = 3 (biological repeats with internal technical repeats for all experiments).

PS1 (hTERT-immortalized) representing normal, healthy PSCs, which, when cultured on plastic, routinely acquire an activated state, was used to represent PSCs (37). As demonstrated before (6), these cells can be rendered quiescent (using ATRA), acquiring a larger shape with changes in canonical markers of PSCs (**Supplementary Figure 1**). LC-MS/MS-based lipid mediator profiling was employed to identify SPMs, using previously defined identification criteria and methods [**Supplementary Figure 2** (41, 45)] in PS1 grown normally [mildly activated: plastic culturing, ethanol 0.1% (v/v): vehicle control] and those rendered quiescent (ATRA 1 µM) and hyper-activated (TGFβ 5 ng/mL, **Figure 1A**) (46). In these PSC (PS1) phenotypic states, we identified SPMs from all four bioactive metabolomes (AA, EPA, DHA, and n-3 DPA) (**Figures 1B–G**; **Supplementary Table 1**) including lipoxins, resolvins, and maresins, in addition to classic eicosanoids [i.e., leukotrienes, thromboxane, and prostaglandins (47)]. Interestingly, lipid mediator concentrations changed significantly with stellate cell activation status. Principal component analysis (PCA, data not shown) and partial least square discriminant analysis (PLS-DA, **Figure 1B**) reveal a good clustering of replicate experiments confirming reproducibility. Evaluation of VIP scores in PLS-DA analysis (**Figure 1C**) identifies that 14 lipid mediators significantly (VIP > 1) contribute to the observed separation of the clusters. Among the differentially regulated lipid mediators, we observed an upregulation of the potent leukocyte chemoattractant LTB_4_ and resolvins (RvE1, RvE3 and RvE4) by TGFβ, whereas ATRA upregulated the concentrations of several SPMs including MaR1, RvT4, and RvD5_n-3 DPA_ along with prostaglandins (PGE_2_, PGF_2a_, and PGD_2_).

Having observed that lipid mediator, and in particular SPM, levels were reflective of cellular activation status in immortalized PSCs, we next queried whether primary patient-derived CAFs, previously sub-categorized pCAFassigner A–D subtypes (12), which respond variably to quiescence acquisition upon treatment with ATRA, were also capable of producing SPMs (note: these are non-immortalized cells with limited passaging ability, restricting technical or biological repeats). Here, we observed that lipid mediators were also produced by CAFs in the same amounts as PS1, though there were differences in the SPM profiles produced by each of these CAF subtypes (**Table 1**; **Supplementary Figure 3**).

**Table 1:** CAF lipid mediator profiling.

		Mediator	PS1 (pg/mL)		CAF C (pg/mL)		CAF B (pg/mL)		CAF A (pg/mL)		CAF D (pg/mL)	
			EtOH	ATRA	EtOH	ATRA	EtOH	ATRA	EtOH	ATRA	EtOH	ATRA
**DHA**	**RvD**	**RvD1**	0.31	0.91	0.30	–	0.10	0.44	0.38	0.38	0.42	0.41
		**RvD4**	0.63	0.21	0.75	0.34	0.22	0.31	0.57	0.52	0.38	0.65
		**RvD5**	0.05	0.20	0.06	0.03	0.18	0.23	0.26	0.22	–	–
		**RvD6**	0.16	0.06	0.15	0.13	0.17	0.29	0.24	0.21	0.14	0.17
	**PD**	**PD1**	–	–	0.09	–	–	–	–	–	0.05	–
		**10S,17S-diHDHA**	0.41	0.38	0.26	0.43	0.44	0.57	0.47	0.55	0.37	0.26
		**22-OH-PD1**	0.10	–	–	–	–	0.07	–	–	–	–
		**17R-PD1**	0.04	–	–	–	–	–	0.04	–	0.08	–
	**MaR**	**MaR1**	2.07	1.10	1.72	–	–	–	1.69	1.28	0.83	0.68
		**MaR2**	–	–	0.30	0.33	–	–	0.49	0.35	0.32	0.28
		**4S,14S-diHDHA**	0.22	0.23	–	0.09	0.13	–	0.29	–	–	0.19
**n-3 DPA**	**RvT**	**RvT3**	–	–	2.81	–	–	–	1.61	2.72	–	–
		**RvT4**	0.05	0.22	0.18	–	0.29	0.29	0.25	0.33	0.16	0.20
	**RvD_n-3 DPA_**	**RvD1_n-3 DPA_**	–	–	–	–	–	–	–	0.15	0.26	0.47
		**RvD2_n-3 DPA_**	–	5.61	–	1.03	–	0.47	–	–	1.56	–
		**RvD5_n-3 DPA_**	–	0.20	0.44	–	0.47	0.48	0.38	0.34	0.21	–
	**PD_n-3 DPA_**	**PD2_n-3 DPA_**	0.19	0.29	–	–	0.42	0.51	1.69	1.65	0.26	0.16
		**22-OH-PD1_n-3 DPA_**	1.19	–	–	–	–	–	–	–	–	–
	**MaR_n-3 DPA_**	**MaR2_n-3 DPA_**	0.94	3.85	–	1.12	1.66	3.27	3.10	1.45	2.86	1.05
		**7S,14S-diHDPA**	–	–	–	–	–	2.78	–	–	–	–
**EPA**	**RvE**	**RvE1**	0.14	–	0.61	–	0.39	0.28	0.56	0.15	–	–
		**RvE2**	–	–	–	–	0.31	0.42	0.42	0.47	–	–
		**RvE3**	1.16	–	0.35	1.20	0.38	1.07	0.35	1.12	1.33	0.68
		**RvE4**	0.42	–	–	0.34	–	–	–	0.25	–	0.73
**AA**	**LX**	**LXB_4_**	18.23	4.09	3.74	15.03	6.93	4.88	3.63	5.37	18.58	22.39
		**5S,15S-diHETE**	9.98	11.91	10.75	5.71	6.15	7.57	9.90	8.69	7.92	8.31
		**13,14-dehydro-15-oxo-LXA_4_**	5.22	2.46	4.32	2.39	1.12	1.42	1.16	0.87	1.32	3.24
		**15-oxo-LXA_4_**	–	–	–	–	0.05	–	–	–	–	–
		**15-epi-LXA_4_**	2.32	1.77	8.34	3.01	1.67	3.02	2.30	4.26	3.19	4.35
		**15-epi-LXB_4_**	–	2.97	–	–	–	–	–	4.51	–	–
	**LTB**	**LTB_4_**	–	–	0.27	0.25	0.30	0.21	–	–	–	–
		**5S,12S-diHETE**	0.12	0.11	0.28	–	–	–	–	0.37	0.11	0.22
		**6-trans-LTB_4_**	0.05	0.15	0.03	0.05	0.14	0.15	0.24	0.27	0.03	0.06
		**6-trans-12-epi-LTB_4_**	0.22	0.18	0.20	0.28	0.43	0.35	0.75	0.63	0.22	–
	**PG**	**PGE_2_**	24.86	156.68	65.74	119.97	28.81	246.44	6.63	33.09	11.39	7.79
		**PGD_2_**	24.46	20.86	91.08	32.99	27.42	40.30	17.36	29.92	41.11	70.45
		**PGF_2a_**	14.44	12.88	8.96	4.70	4.75	9.02	4.10	5.91	1.27	0.63
	**TX**	**TxB_2_**	6.64	1.99	16.38	7.09	5.26	5.75	5.48	5.27	4.96	6.44

Patient-derived cancer-associated fibroblasts (CAFs) and PS1 cell culture lipid mediator concentrations (n = 1). (-) below limits of detection.

To better understand the inter-relationships involved in SPM synthesis after PSC activation/quiescence, a network pathway analysis was performed (**Figures 1D–G**). Since the production of most of these mediators involves the coupling of ALOXs and COX for the sequential oxygenation at different carbon atoms within the fatty acid backbone, network analysis allows for an easier visualization of the mediators (same pattern informs of biosynthetic pathway), the enzymes involved in their synthesis grouped by common precursors (AA, EPA, DHA, or n-3 DPA), and allows deciphering of common pathways being activated (colored), providing insights into how they may be regulated by the treatment (mini heatmaps). This analysis indicated that incubation of PSC with TGF-β preferentially upregulated the activity of ALOX5 in these cells and the conversion of eicosanoids, namely, AA and EPA, to yield LTB_4_, RvE1, RvE3, and RvE4. Intriguingly, incubation of PSCs with ATRA, which results in a quiescent phenotype, was observed to upregulate the activity of ALOX12 (MaR1, 7S,14S-diHDHA, and 4S,14S-diHDHA), ALOX15 (RvD6 and RvD5_n-3 DPA_), and COX-2 (RvT4). Taken together, these findings suggest that PSC/CAF lipid mediator profiles in pancreas reflect their activation status. Therefore, we next explored the mechanisms for the change in PSC SPM profile upon quiescence mediated by ATRA since this is therapeutically tractable in reducing tumor invasion (5–7).

### 3.2 Lipid mediator biosynthetic enzymes and cognate receptors are spatially regulated upon PSC quiescence

**Figure 2:**
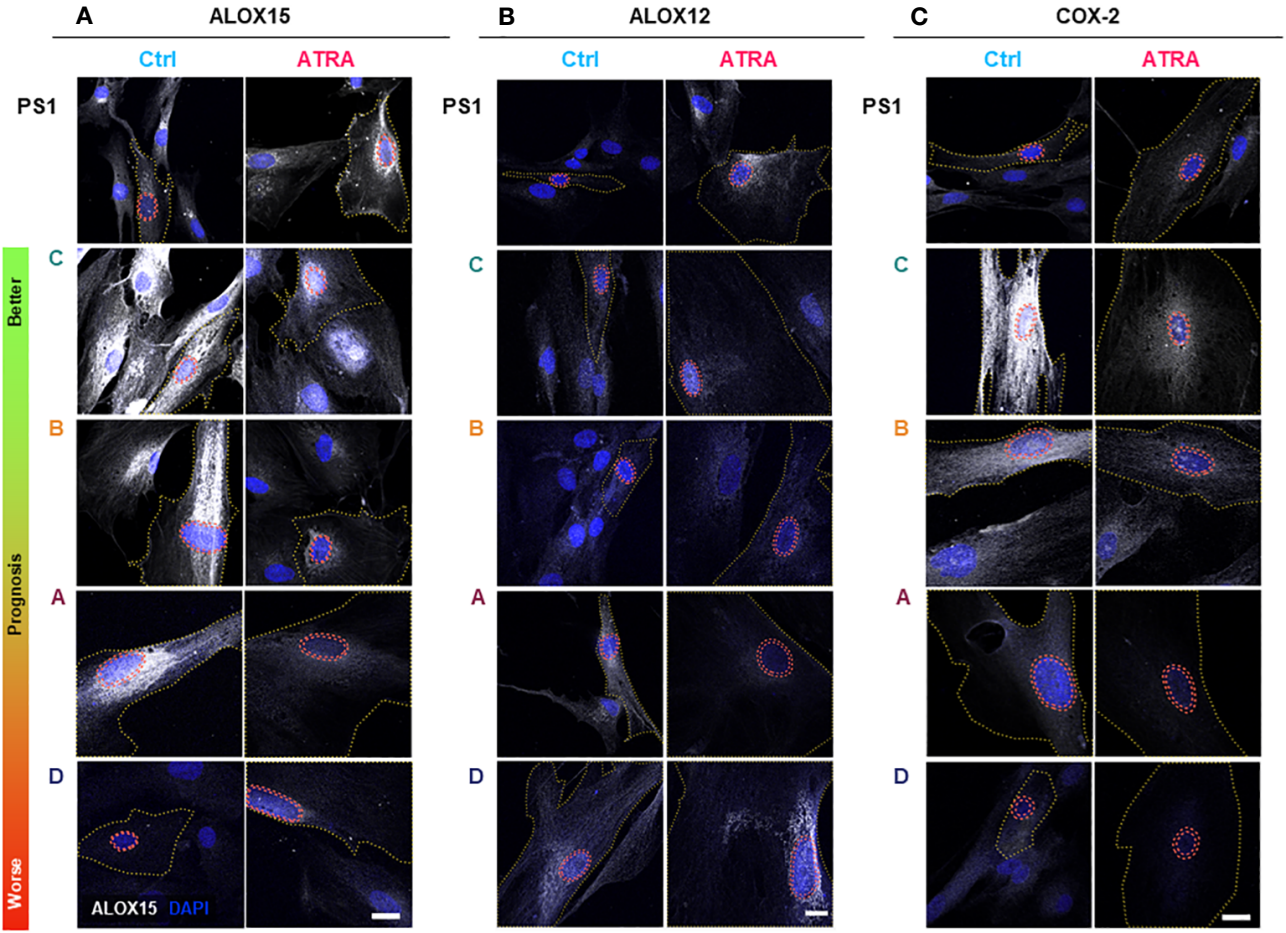
PSC and cancer-associated-fibroblast (CAF) lipid mediator synthetic enzyme expression and subcellular localization in quiescent and activated state. PSC (PS1) and CAFs previously sub-categorized into four groups (A–D, according to Neuzillet 2019 (12) and herein ordered by prognostic relevance: C, B, A, and D, from better to worse prognosis) were cultured on coverslips, treated with ATRA 1 µM daily for 7 days. Representative immunocytochemistry images for ALOX15 **(A)**, ALOX12 **(B)**, and COX-2 **(C)** expression in ATRA-treated PSC/CAF cells (red) compared to vehicle controls (blue). Respective IgG (control) antibodies were used for background setting (**Supplementary Figures 5**–**7**). Red dashed tram lines mark nuclear envelope. The yellow dotted line depicts cell boundaries defined by concurrent αSMA co-staining (not shown for simplicity). ALOX15, ALOX12, and COX2 are shown in grayscale (white color) and DAPI is shown in blue. Scale bar = 20 µm. ns, not significant.

**Figure 3:**
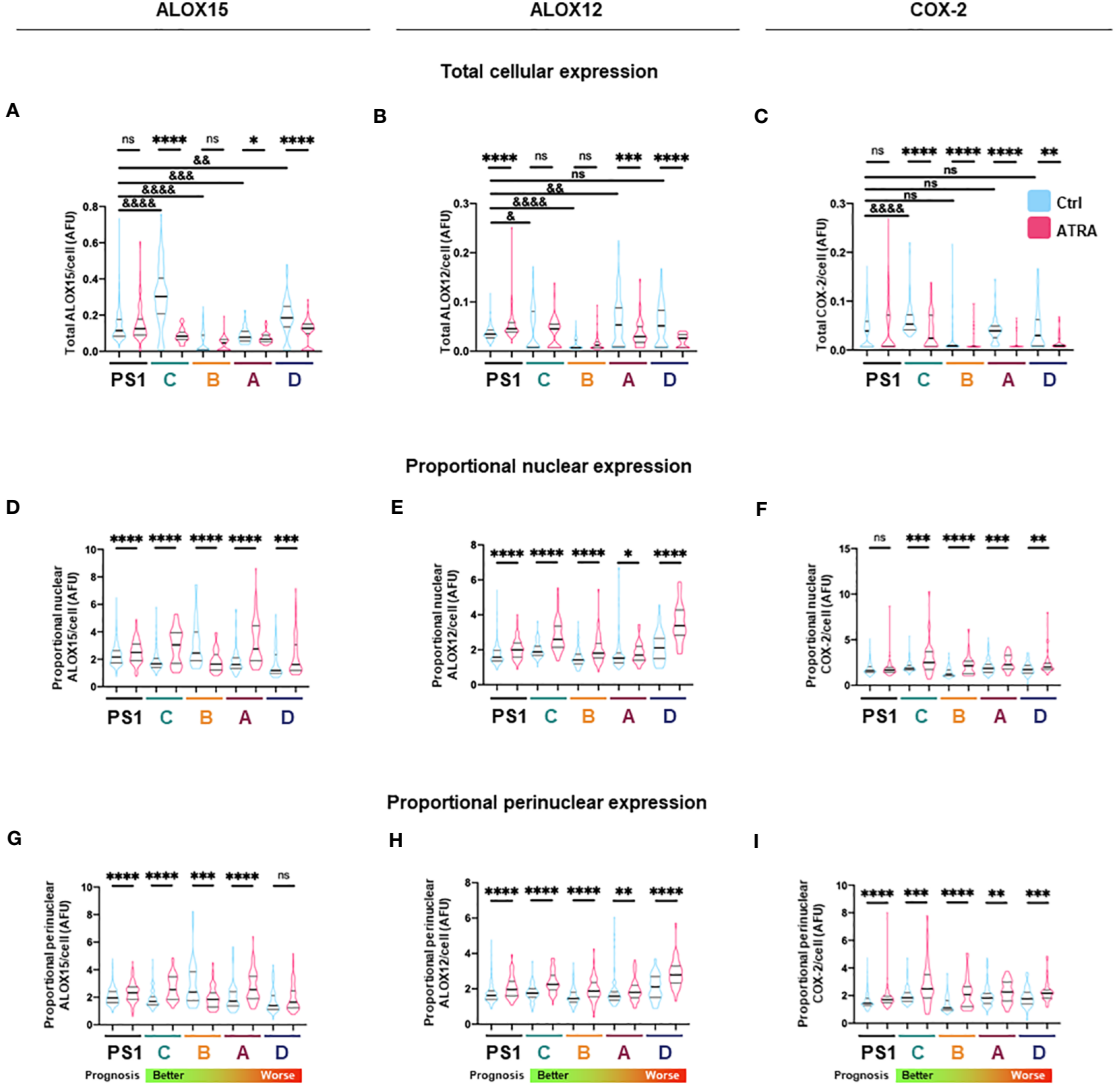
Total and subcellular lipid mediator biosynthetic enzyme expression in PSC and cancer-associated-fibroblasts (CAFs) in different activation states. **(A–C)** Quantification of total cellular expression (median intensity) by immunocytochemistry for ALOX15, ALOX12, and COX2 from three independent experiments (median ± IQR) in PSC (PS1) and CAF (subtypes C, B, A, D: ordered from better to worst patient prognosis). **(D–F)** Quantification of proportion of nuclear ALOX15, ALOX12, and COX2 expression as defined by ratio of nuclear to total cellular median intensity delineated by DAPI edge (median ± IQR). **(G–I)** Quantification of proportion of nuclear envelope ALOX15, ALOX12, and COX2 expression as defined by ratio of perinuclear space to total cell median intensity where perinuclear space area was determined by expanding and shrinking DAPI edge by 1 µm (2 µm in total, red dashed lines). Mann–Whitney against same cell-type vehicle control (*) or against PS1 vehicle control (^&^). *, ^&^*p* < 0.05, **, ^&&^*p* < 0.01, ***, ^&&&^*p* < 0.001, ****, ^&&&&^*p* < 0.0001. AFU, arbitrary fluorescent units.

Since SPM production is dependent on expression (48) and subcellular localization of SPM biosynthetic enzymes (49–51), we explored the relevant enzymes [arachidonate lipoxygenases (ALOX5, ALOX12, and ALOX15) and cyclo-oxygenases (COX-1 and COX-2)] in PSC and CAF subtypes upon ATRA treatment (**Figures 2**, **3**). There appears to be minimal, if any, change in the transcript expression of these enzymes within the PSC cell line (PS1) or primary human CAFs (all subtypes A–D) upon treatment with ATRA, except for a uniform downregulation of COX-1 (**Supplementary Figure 4**). These observations rule out a transcriptional control of biosynthetic enzymes by ATRA and corroborate with our previous transcriptional gene expression data [*PTGS1*, when PSCs were treated with supra-physiological 10 µM of ATRA (6)].

We next looked at protein expression of biosynthetic enzymes in order to elucidate how the differential lipid mediator profiles observed are being regulated. ALOX15, ALOX12, and COX-2 (**Supplementary Figures 4**–**7**; **Figure 2**) were differentially expressed at the basal (untreated) level in the PSC cell line (PS1) and primary human CAFs (subtypes A–D) as shown by quantification from immunofluorescence total cellular levels (**Figures 3A–C**). In PSC (PS1 cells), total ALOX12 expression is increased upon treatment with ATRA at the more physiologically relevant 1 µM daily for 7 days (**Figure 3B**), whereas total ALOX15 and COX-2 remain unchanged as defined by total cell intensity (**Figures 3A, C**). Interestingly, within most CAF subtypes (A–D), the total cell intensity in ALOX15 is reduced upon treatment with ATRA (**Figure 3A**). On the other hand, ALOX12 expression is downregulated in CAF subtypes A and D following treatment with ATRA, whereas the expression of this enzyme is upregulated in PSC (PS1) (**Figure 3B**). Lastly, COX-2 expression is downregulated in all CAF subtypes (A–D) following ATRA treatment (**Figure 3C**), while it remains unchanged for PSC (PS1). These results, at least in part, explain the differences in production of SPMs in normal PSC from healthy pancreas and different CAF subtypes, which are educated by cancer cells (**Figure 1**; **Table 1**).

We next explored the subcellular localization of these biosynthetic enzymes, since this aspect is linked to a switch in their product profile (49–52). Utilizing state-of-the-art cell-profiling image analysis methods, we quantified nuclear-to-total cell intensity ratio (**Figures 3D–F**) as well as the perinuclear space (indicating nuclear envelope)-to-total cell intensity ratio (**Figures 3G–I**) as a surrogate marker of enzyme translocation upon ATRA treatment. We demonstrated that ALOX15, following treatment with ATRA, moves into the nucleus and the perinuclear space for all PSC and all CAF subtypes except CAF B. In contrast, ALOX12 expression is universally across all PSC and CAF subtypes, observed around the nucleus and perinuclear space upon ATRA treatment. COX-2 follows the same nuclear translocation pattern as ALOX12 in all CAF subtypes, whereas its expression remains unchanged in PSC (PS1) cells (**Figure 3F**). This subcellular localization of various enzymes associated with ATRA treatment suggests a spatial shuttling of key elements such as substrates and enzymes that may regulate the differential expression of SPM in healthy PSCs and primary human CAFs derived from cancers.

GPCRs are increasingly recognized to be central in mediating the biology of SPM. GPR101 is the cognate receptor for RvD5_n-3 DPA_ (53), GPR18/DRV2 for RvD2 (53), GPR37 for PD1 (54), and FPR2/ALXR for LXA_4_, RvD1, and RvD3 (55) (**Supplementary Figure 8A**). GPR32 (receptor for RvD1 (56), LXA_4_, 15-epi-LXA_4_, RvD3, AT-RvD3, and RvD5), LGR6 (receptor for MaR1), and ChemR23 [receptor for RvE1 (57)] were not expressed in PSCs and CAFs (data not shown). GPR37 has significantly lower transcript level expression in CAF compared to PSC. BLT-1 was downregulated in in CAF subtypes C and A whereas ALXR was found upregulated in CAF subtype C as compared to PSCs.

In contrast, GPR101 mRNA expression was upregulated in PSCs upon ATRA treatment (**Supplementary Figure 8B**), resulting in the possibility that quiescent PSC may be more responsive to RvD5_n-3 DPA_ (53). Since RvD5_n-3 DPA_ production also increased in PSC upon ATRA treatment (**Figure 1**), the upregulation of its cognate receptor GPR101 (53) might suggest a positive feedback loop to sustain the phenotype or function. This upregulation of GPR101 was not observed in any of the CAF subtypes, probably suggesting that cancer education may ameliorate this responsiveness.

It is known that various tumors express ALOX5 and COX-2 (58). Therefore, in order to understand if cancer cells could synthesize and/or respond to SPMs, we sought to determine if two representative diverse pancreatic cancer cell lines (AsPC-1 and MIAPaCa-2) expressed the SPM biosynthetic enzymes and receptor transcripts. Several enzymes and receptors were found to be transcriptionally expressed at higher levels than PSCs except for COX-1 (**Supplementary Figures 9A, B**). We next investigated cancer cell–PSC interactions.

### 3.3 RvD5_n-3 DPA_ is an important factor in cancer cell invasion

Having observed that RvD5_n-3 DPA_ and its receptor GPR101 are upregulated by ATRA, we next investigated the role of this mediator in regulating cancer invasion. For this purpose, we used two distinct 3D physio-mimetic co-culture models with representative surrounding ECM to study cancer cell and CAF invasion (organotypics and spheroids) (39, 40).

**Figure 4:**
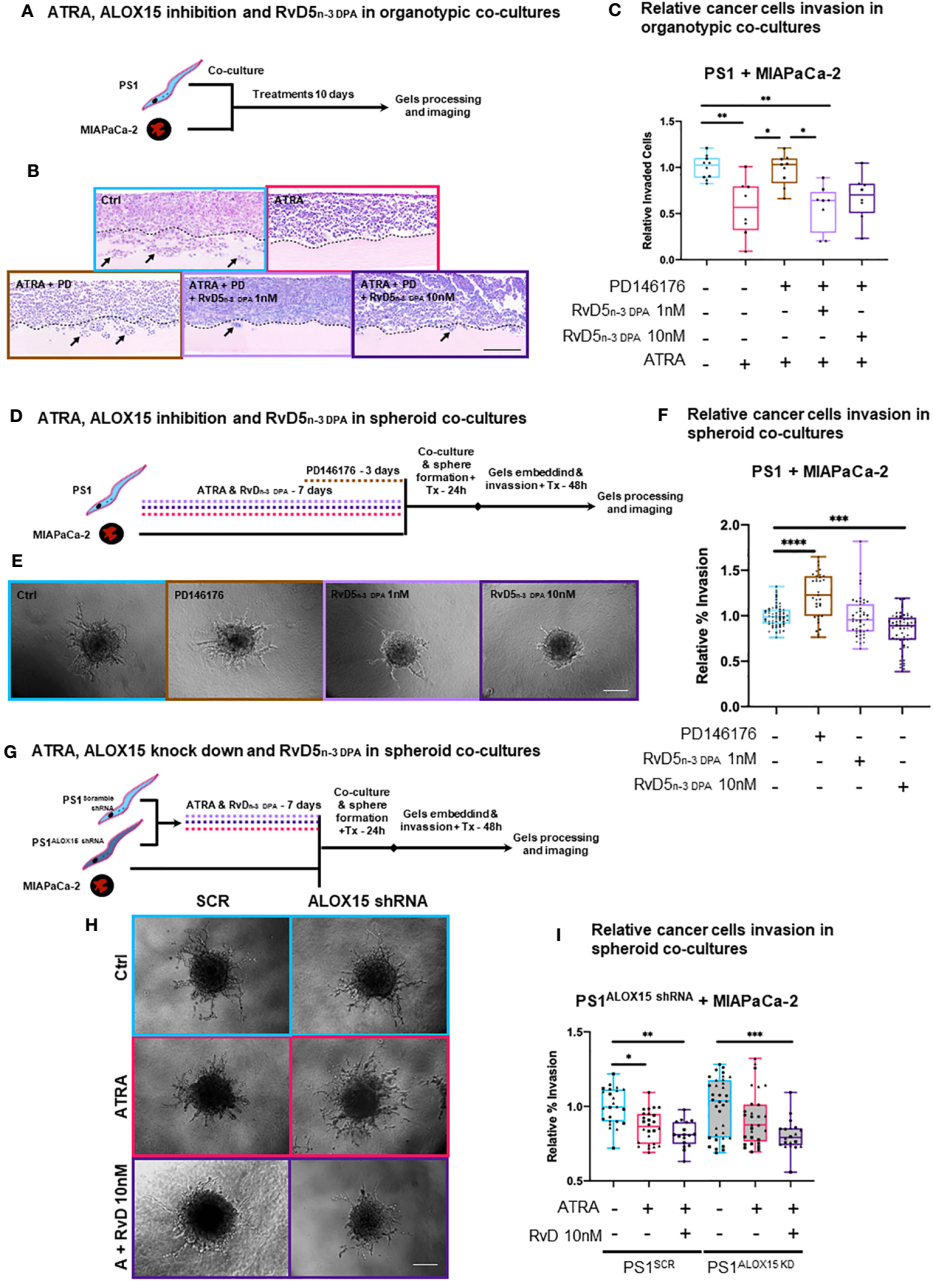
3D *in vitro* physio-mimetic (organotypic and spheroid) invasion assays after ALOX15 inhibition [chemical (PD146176) and genetic (shRNA)]. **(A)** Schematic of PSCs (PS1) and cancer (MIAPaCa-2) cells were co-cultured at a 2:1 ratio, respectively, in an organotypic 3D invasion assay and treated daily with ATRA 1 µM, vehicle control (Ctrl, ethanol 0.1%), PD146176 5µM, and/or RvD5_n-3 DPA_ 1/10 nM for 10 days while allowing invasion. **(B)** Representative organotypic 3D invasion assay H&E images for each of the conditions. The dotted line represents the boundary between ECM gel and the cell layer from where invasion (marked by arrows) is counted. Scale bar = 300 µm. **(C)** Relative (normalized to vehicle control) invaded cell quantifications with each data point reflects the median value of the number of invaded cells for five serial 30× fields (limited to the area of cellularity avoiding edge artifacts) per one gel. Different symbols indicate technical replicates (2–6) within biological replicates (*n* = 3). **(D)** Experimental schematic of PSCs pre-treated with either ATRA (7 days), PD146176 5 µM (3 days), and/or RvD5_n-3 DPA_ 1 or 10 nM (7 days) and then co-cultured with cancer cells to form spheres and subsequently embedded in Matrigel/collagen gels and allowed to invade for 2 days. **(E)** Representative 20× bright field microscopy images of hanging droplet spheroid invasion assay model for each of the conditions. Scale bar = 200 µm. **(F)** Relative (normalized to respective vehicle control) invaded cell quantifications (%invasion derived from invaded area divided by central spheroid area). Different symbols indicate technical replicates (2–6) within biological replicates (*n* = 3). **(G)** Representative 20× images of the hanging droplet spheroid invasion assay model. ALOX15 shRNA knockdown PSCs or respective controls were pre-treated with either ATRA (7 days) or RvD5_n-3 DPA_ 10nM (7 days) and then co-cultured with cancer cells to form spheres and subsequently embedded in Matrigel/collagen gels and allowed to invade for 2 days. Scale bar = 200 µm. **(H)** Representative 20× bright field microscopy images of hanging droplet spheroid invasion assay model for each of the conditions. **(I)** Relative (normalized to vehicle control) invaded cell quantifications (%invasion derived from invaded area divided by the central spheroid area). Different symbols reflect technical replicates (2–6) within biological replicates (*n* = 3). Kruskal–Wallis with Dunn’s multiple comparisons test. *p < 0.05, **p < 0.01, ***p < 0.001, ****p < 0.0001.

We first employed a pharmacological approach to inhibit RvD5_n-3 DPA_ biosynthesis. Using an organotypic model, PSCs are co-cultured and treated simultaneously with ATRA [to induce quiescence (6)] or PD146176 [an ALOX15 inhibitor (59), the initiating enzyme in RvD5_n-3 DPA_ biosynthesis (29)] as cells interact and invade into the gel. ATRA reproducibly reduces the number of invading cancer cells into the gel (**Figures 4A–C**) (6, 7). In contrast, upon ALOX15 inhibition (PD146176) at the same time as ATRA treatment, this reduction in invasion is abrogated, supporting a potential role for RvD5_n-3 DPA_ in the observed activities of ATRA (**Figure 1**). Of note, addback of RvD5_n-3 DPA_ [1 nM, a concentration that was selected based on known dissociation constant of the mediator to GPR101 (53)] in cells treated with the ALOX15 inhibitor rescued the protective activities of ATRA (**Figures 4A–C**; **Supplementary Figures 10A, B**). Taken together, this suggests that the effect of ATRA in reducing invasion is, at least in part, mediated by RvD5_n-3 DPA_.

To investigate if the observed effect of RvD5_n-3 DPA_ in the organotypic models was mediated exclusively by PSCs, we next employed a spheroid model, where only PSCs are pre-treated and then co-cultured with cancer cells to form spheres and subsequently allowed to invade. Under these conditions, we observed that inhibition of ALOX15 significantly increased invasion and that RvD5_n-3 DPA_ reduced invasion (**Figures 4D–F**). Although ATRA did not show a significant reduction at this time in the spheroid model (possibly due to shortened interaction time -frame, **Supplementary Figures 10C, D**), a trend in reduction can be observed. When the ALOX15 inhibitor was added to ATRA, invasion significantly increased as compared to ATRA alone (**Supplementary Figures 10C, D**). Notably, addback of RvD5_n-3 DPA_ to ATRA and the inhibitor incubation abrogate the inhibitor-mediated invasion (**Figures 4D–F**) and these experimental conditions did not change proliferation of PSCs or cancer cells (**Supplementary Figure 11A**).

To further explore the role of RvD5_n-3 DPA_, we used an shRNA approach to abrogate the expression of ALOX15 using a lentiviral shRNA system (**Supplementary Figure 11B**). In this experimental setup, in the scramble shRNA (SCR) control PSCs being co-cultured with cancer cells, ATRA reduced the invasion area as compared to vehicle control (**Figures 4G–I**). Interestingly, when ALOX15 was knocked down, ATRA-mediated reduction in invasion was lost and rescued by the addition RvD5_n-3 DPA_ (**Figures 4G–I**).

Taken together, these results from independent and distinct co-culture experiments indicate that, indeed, ATRA-mediated quiescence in PSC, which, in turn, reduces cancer cell invasion by paracrine effects, is at least in part due to PSC-derived RvD5_n-3 DPA_, an observation that opens up the field of paracrine lipid mediator (SPM) signaling, which has been hitherto not studied in cancer–stroma interactions. This needs further interrogation in more complex *in vitro* and *in vivo* systems that include other stromal components such as immune cells.

### 3.4 SPMs can differentiate stage IV PDAC patient plasma versus healthy controls

**Figure 5:**
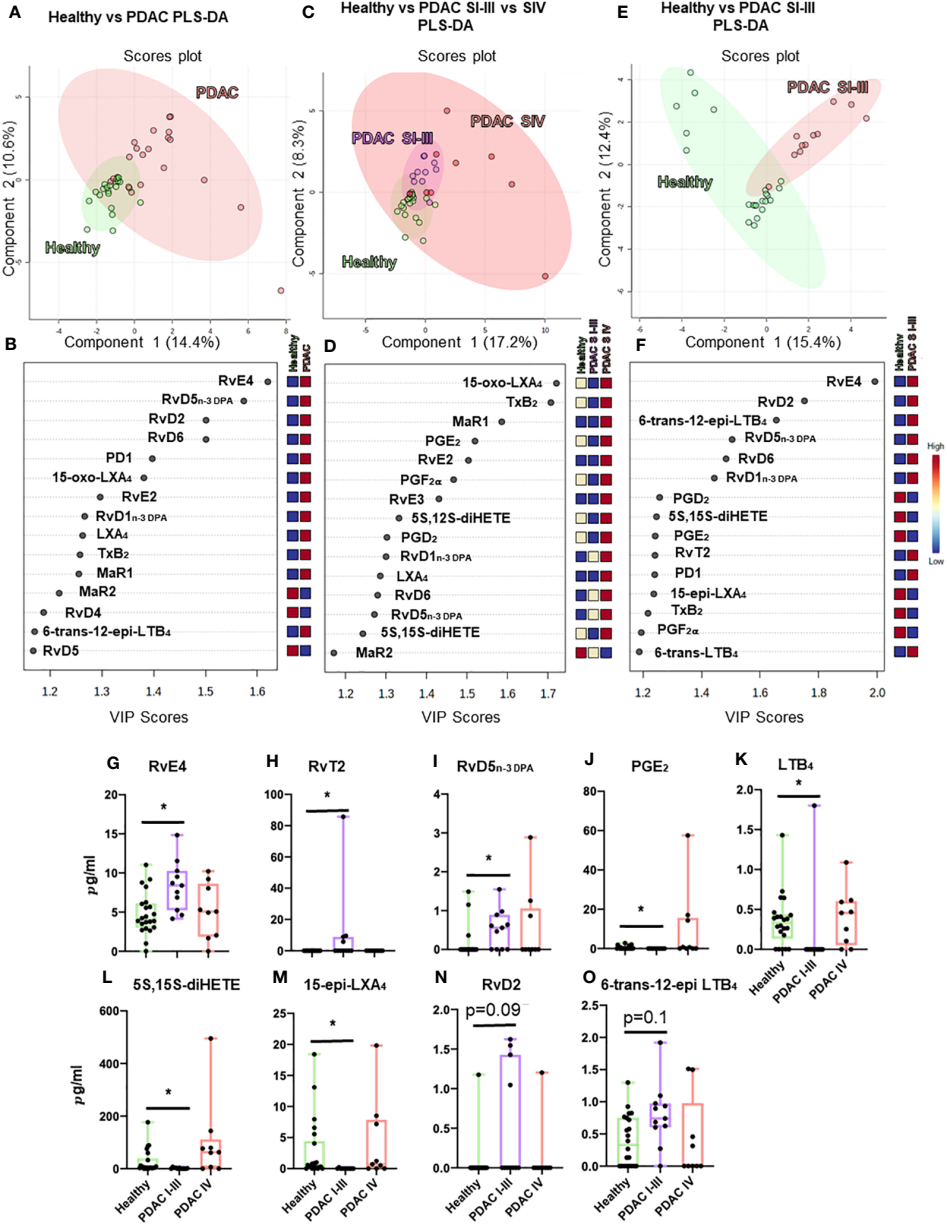
Plasma lipid mediator profiling from patients with PDAC and healthy volunteers. Summary data from LC-MS/MS-based lipid mediator profiling using plasma from a cohort of 20 PDAC patients and 22 matched healthy volunteers resolved by normalized values using multivariate partial least squares discriminant analysis (PLS-DA) analyses **(A–F)** to understand clustering and differentiation according to lipid mediators as well as by absolute concentration values **(G–O)**. Multivariate analyses, top: scores plot **(A, C, E)**, bottom: VIP scores **(B, D, F)**, left **(A, B)** PDAC (*n* = 20) vs. healthy volunteers (*n* = 22), center **(C, D)** non-metastatic PDAC (stages I–III, *n* = 11) vs. metastatic PDAC (stage IV, *n* = 9) vs. healthy volunteers (*n* = 22), and right **(E, F)** non-metastatic (*n* = 11) vs. healthy volunteers (*n* = 22). **(A–I)** Graphs represent differences observed in significantly regulated mediators (FDR-corrected *p*-value *<0.05 Mann–Whitney *U*-test). Zero values represent values below the detection limit for LC-MS/MS.

**Figure 6:**
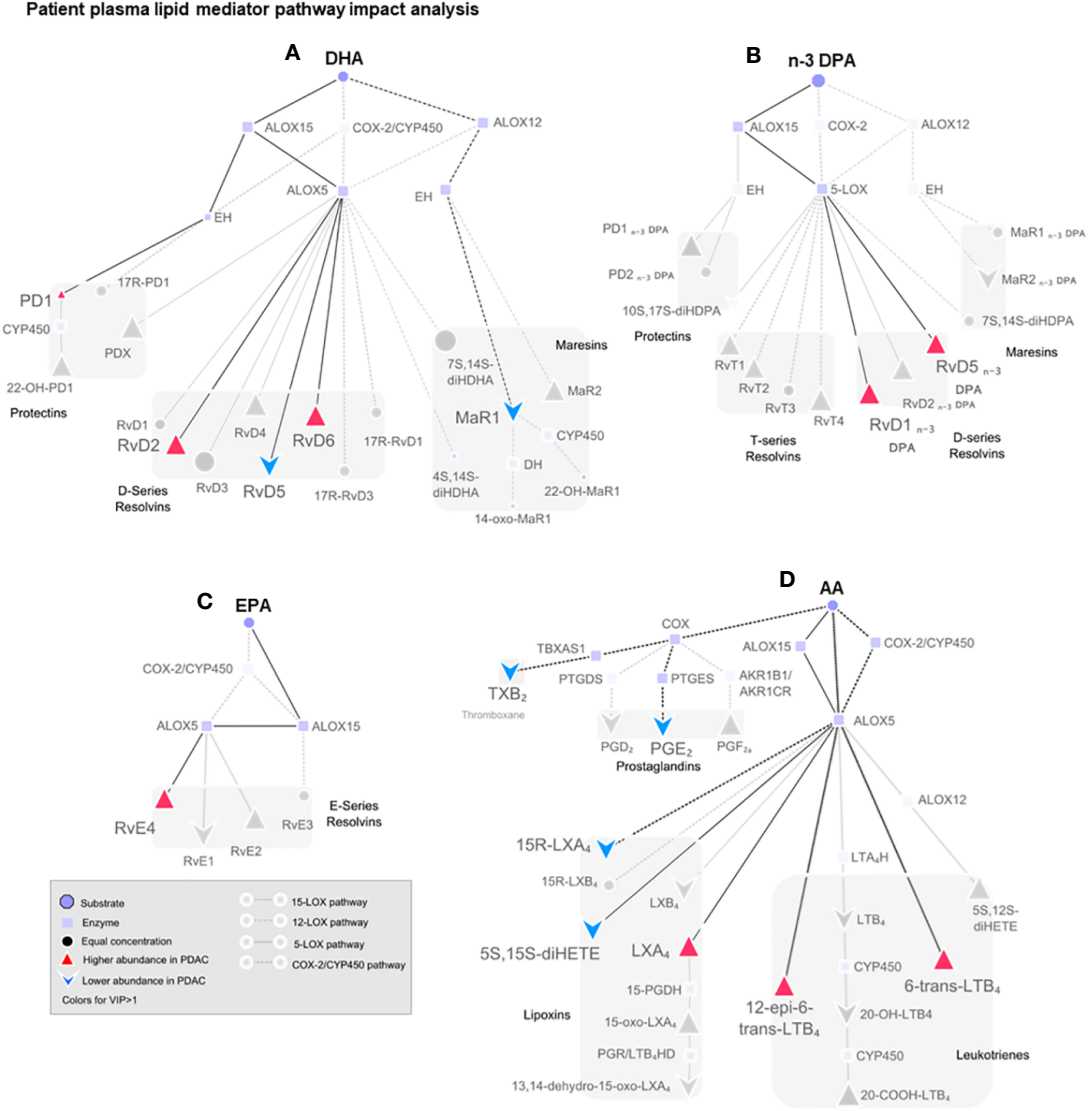
Pathway analysis of SPM production in human plasma from healthy volunteers and patients with PDAC. Pathway analysis on non-metastatic PDAC patients against healthy volunteers highlighting (in color) the mediators that were found differentially regulated in PLS-DA VIP scores >1 and the synthetic pathways highlighted in dark. Dull dots represent mediator not detected. Each synthetic pathway is demonstrated with a distinct connecting line. Figure is split according to lipid mediator metabolomes: **(A)** docosahexaenoic acid (DHA), **(B)** n-3-docosapentaenoic acid (n-3 DPA), **(C)** eicosapentaenoic acid (EPA), or **(D)** arachidonic acid (AA). PDAC (*n* = 20), healthy volunteers (*n* = 22).

Having observed a role for RvD5_n-3 DPA_ in regulating aspects of pancreatic cancer progression, we hypothesized that patients with PDAC would exhibit a different SPM profile compared to healthy volunteers. Since it is not possible to obtain pancreatic tissues from healthy volunteers, we explored if the circulating plasma RvD5_n-3 DPA_ together with that of other SPMs were altered in PDAC patients when compared with age- and gender-matched control cohorts. We ensured that patients and controls were not on NSAIDs and anti-diabetic medications, and samples were obtained before any cancer-directed treatment was started. Using LC-MS/MS-based lipid mediator profiling, we identified mediators from all four essential fatty acid families (AA, EPA, DHA, and n-3 DPA; **Figures 5**, **6**) (45).

Multi-variate PLS-DA analysis of the identified lipid mediators indicated that concentrations of these molecules in PDAC plasma were distinct to those from healthy volunteers as illustrated by a separation in the clusters representing lipid mediator concentrations for each group (**Figure 5A**). VIP scores identified several SPMs, pathway markers, and further SPM metabolites to be markedly different between the two groups with most SPMs being observed at higher concentrations in patients with PDAC (**Figure 5B**). These observations suggest that lipid mediator pathways are engaged in these patients. PDAC patients were then sub-clustered as metastatic (stage IV, SIV) and non-metastatic (stages I to III, SI–III) from which PLS-DA analysis performed well at discriminating the groups (**Figures 5C–F**). When metastatic patients were excluded from multivariate analysis, we observed a better separation between the different clusters when using both supervised (PLS-DA) and unsupervised (PCA) multivariate analysis (**Figures 5E, F**; **Supplementary Figures 12A–C**). Evaluating plasma concentrations of individual SPM in patients with non-metastatic PDAC demonstrated significantly higher (FDR-adjusted *p* < 0.05) concentrations of RvE4 (**Figure 5G**), RvT2 (**Figure 5H**), and RvD5_n-3 DPA_ (**Figure 5I**) and lower PGE_2_ (**Figure 5J**) and LTB_4_ (**Figure 5K**) levels when compared with those from healthy controls. We also observed that RvD5_n-3 DPA_ concentrations were markedly reduced in peripheral blood from patients with metastatic PDAC when compared with those observed in non-metastatic PDAC patients. Network pathway analysis was also carried out to better understand the regulation of lipid mediator biosynthetic pathways in patients with non-metastatic PDAC compared to healthy individuals (**Figure 6**). Our analyses suggest that ALOX5 and ALOX15 pathways are upregulated in patients with non-metastatic PDAC, while ALOX12 and COX pathways are downregulated in peripheral blood, contributing to the observed regulation of differences in lipid mediator profiles.

## 4 Discussion

Activated PSCs and/or CAFs have been increasingly considered orchestrators within the PDAC microenvironment aiding tumor establishment, growth, and migration. Thus far, our total focus has been placed on protein-based signaling in evaluating this molecular cross-talk (60). In this report, for the first time, we explore lipid mediators, mainly SPMs, using state-of-the-art LC-MS/MS, to demonstrate that they are key components in this molecular cross-talk.

Since prostaglandins regulate immune cell function (61), we hypothesize that quiescent PSCs may act as immune homeostatic modulators by producing prostaglandins and SPMs. However, in the context of the PDAC microenvironment, characterized by PSC activation, a switch in autacoid signaling occurs similar to well-understood secretion of various cytokines (IL-1, 6, 8, 10, and MCP-1) (60). Thus, the production of RvEs, in conjunction with cytokine signaling, may perhaps activate a self-limited and physiological inflammatory response to determine both the magnitude and duration of the response (31, 62, 63). Interestingly, among the many SPMs found to be regulated with the activation of PSC and, also noted, in the plasma of patients with PDAC, we explored RvD5_n-3 DPA_ in more detail. We demonstrate using physio-mimetic *in vitro* models specifically designed to dissect cancer-fibroblast/stellate cell interaction that, in fact, RvD5_n-3 DPA_ could have a fundamental role in PDAC biology by mediating PSC-led cancer cell invasion.

Recent studies demonstrate that RvD5_n-3 DPA_ regulates key physiological processes to maintain homeostasis, whereby, in the gut, it is important in regulating intestinal epithelial barrier permeability (64). Downregulation of RvD5_n-3 DPA_ together with related resolvins (RvD1_n-3 DPA_ and RvD2_n-3 DPA_) has been linked to increased cardiovascular risk in humans mediated by peripheral blood neutrophil, monocyte, and platelet activation (65), indicating that RvD5_n-3 DPA_ may be systemically important. Locally, RvD5_n-3 DPA_ has been found to limit joint and gut inflammation during inflammatory arthritis (53). Mechanistically, *in vivo* and *in vitro* studies have shown that RvD5_n-3 DPA_ regulates leukocyte trafficking to the site of inflammation, increased bacterial phagocytosis by neutrophils and macrophages, and accelerated the resolution of infectious inflammation (53, 65).

We also suggest a role for receptor regulation, in a CAF-subtype-specific manner, explaining the PDAC microenvironment spatial and temporal heterogeneity (11, 12). These findings open the door for exciting new avenues, other than protein-based signaling, to explore disease mechanisms within the PDAC tumor microenvironment and for the development of targeted therapeutics.

In several experimental tumor resection models, when surgical inflammatory response is blocked pre-operatively, but not post-operatively, with either ketorolac (preferentially inhibits COX-1, and therefore not seen by other NSAIDs) or supplementing SPMs, one could completely abrogate the appearance of micro-metastases or dormancy escape, resulting in long-term survival (34). This effect was mediated by a specific blockade of COX-1 while retaining COX-2 activity. Interestingly, we show that in the PSC/CAF, ATRA induces a universal downregulation of COX-1 RNA, while translocating COX-2 protein expression to the peri-nuclear space. In addition, we studied the expression of enzymes involved in SPM biosynthesis including cyclooxygenases and lipoxygenases in PSCs and CAFs to explore whether differences observed in lipid mediator concentrations were linked to changes in the expression of these proteins. Here, we observed the differences in the subcellular localization of these enzymes between the different cell types, suggesting that changes in the localization of these SPM biosynthetic enzymes may contribute to the observed difference in lipid mediator levels. These observations are in accord with published literature on the importance of subcellular localization in determining the lipid mediator product profile for these enzymes (51, 66). The precise role of this mechanism in SPM production by these cells needs to be explored in future work. Together with differences in the expression of cognate SPM receptors, the present findings suggest a complex interplay in the regulation of tumor progression, which will also need to be further investigated to enable the use of specific SPMs in the management of PDAC.

The present studies uncover a role for SPMs in PDAC pathophysiology as we observed the formation of many of these molecules in this PSC/CAF-centered investigation. For example, RvE4 (in addition to RvD5_n-3 DPA_) was elevated whereas LTB_4_ and PGE_2_ were reduced in non-metastatic treatment-naïve patients with PDAC, compared to healthy volunteers. This distinction is lost upon the appearance of visible metastasis, which may overwhelm SPM and inflammation pathways. In this report, SPMs differentially expressed in patients with non-metastatic PDAC compared to healthy controls have also been found to be regulated by the activation status of PSCs/CAFs *in vitro*. These observations may imply that the circulating SPMs in patients with PDAC originate in the tumor stroma, but it is possible that SPMs may also arise from the tumoral compartment, a systemic response in the vasculature endothelium, or the local or systemic immune system. Even though further studies are warranted to dissect the source in multicellular models, our findings indicate the potential of these findings to be developed as disease biomarkers.

These early observations of resolution pathways being downregulated at severe illness suggest that when the disease burden is excessive or not contained within the local pancreatic microenvironment, the inflammation resolution program may be overwhelmed, leading to exhaustion. In fact, we recently observed a similar phenomenon with COVID-19 patients, where mild disease patients showed an alteration of SPM pathways. However, in severe patients, the system becomes saturated and SPM production becomes impaired (41). Whether this shift happens at the micro-metastatic stage or whether it can be reversed on tumor removal will need further investigation. In a recent study, using immunosorbent assays rather than the more stringent LC-MS/MS, patients with PDAC demonstrated elevated RvD1, RvD2, LXA_4_, and LXB_4_ but with no differences when stratifying by TNM stages (36). Thus, SPM biology may have important spatial and temporal role in PDAC progression.

In summary, our results reveal an exciting novel biology in SPM biosynthesis regulation in PSC/CAF, which is very relevant to further understand PDAC pathophysiology and exploit these signals for therapeutic and diagnostic benefit by conducting more in-depth and wider studies using different model systems and a larger cohort of patients, and in the context of clinical trials such as STARPAC2 (ClinicalTrials.gov Identifier: NCT04241276) where stroma is therapeutically targeted. STARPAC2 utilizes ATRA as a stromal modifying agent in conjunction with standard chemotherapy. Using PTX3, as one of the stromally derived biomarkers, we have already demonstrated that inflammatory mediators expressed by stromal cells may have diagnostic and predictive benefit (5, 8). Furthermore, many other clinical trials in different cancers are underway to modulate inflammation as an adjunct to treat cancer or prevent its recurrence.

## Data availability statement

The datasets presented in this study can be found in online repositories. The names of the repository/repositories and accession number(s) can be found below: https://www.ebi.ac.uk/biostudies/studies/, S-BSST856 https://www.ebi.ac.uk/biostudies/studies/, S-BSST859 https://www.ebi.ac.uk/biostudies/studies/, S-BSST860.

## Ethics statement

The studies involving humans were approved by the Barts Pancreas Tissue Bank (REC approval: 18/SC/0630, projects 2019/12/QM/HK/P/Blood, 2020/06/QM/HK/E/Blood). The studies were conducted in accordance with the local legislation and institutional requirements. The participants provided their written informed consent to participate in this study.

## Author contributions

GA: Performed experimental work, analyzed data, designed the experiment, and wrote and approved the manuscript. MG: Performed experimental work and approved the manuscript. JD: Study design, intellectual advice, and wrote and approved the manuscript. HK: Study design, intellectual advice, arranged funding, and wrote and approved the manuscript. Members of Barts Pancreas Tissue Bank provided material and approved the manuscript. All authors contributed to the article and approved the submitted version.

## Group member of Barts Pancreas Tissue Bank

Christine Hughes, Ahmet Imrali, Rhiannon Roberts, and Amina Saad.

## Supplementary material

Supplementary Figure 1 PSC phenotype characterization and quiescence. Representative immunocytochemistry images of fixed PS1 cells on coverslips stained for **(A)** vimentin, **(B)** GFAP, **(C)** Desmin and **(D)** α-SMA all shown in green after vehicle control (Ctrl, ethanol, 0.1%) or ATRA (1µM) treatment. **(E-H)** Quantification of mean pixel intensity per coverslip from independent biological replicates (n=3, technical replicates represented by different shapes). Each data-point represents median value from same coverslip of 5-10 cells per coverslip for ATRA (right column, red) and vehicle control (left column, blue) with summary data as median and interquartile range. Mann-Whitney U test. ****p*<0.001, ns = not significant. Scale bar: 100µm. Representative Western blots for PSC markers **(I)** vimentin, **(J)** GFAP, **(K)** Desmin and **(L)** α-SMA, after ethanol (vehicle control, Ctrl) or ATRA treatment along with quantification **(M-P)** for each marker normalized to HSC70 and to vehicle control from three independent experiments (each dot) with summary data as mean +/- SD.

Supplementary Figure 2 Representative MRM chromatograms for identified lipid mediators of inflammation. **(A-F)** Multiple reaction monitoring (MRM) chromatogram method was developed with signature ion fragments (mass/charge, m/z) for each molecule monitoring the parent ion (Q1) and a characteristic daughter ion (Q3) (transitions). Identification was conducted using a minimum of six diagnostic ions in total ion count EPI spectrum, MRM peaks with AUC above 2000 conformed by more than five data points and a signal (blue)-to-noise (pink) ratio (S/N) above 3. Representative data are shown for PGE_2_ **(A),** LTB_4 _**(B),** RvE4 **(C),** RvD6 **(D),** MaR1 **(E),** RvD5_n-3DPA _**(F)**.

Supplementary Figure 3 Comparison of lipid mediators secreted by pancreatic stellate cells and cancer associated fibroblasts (along with sub-types). Patient-derived cancer associated fibroblasts (CAFs) previously sub-categorized into four groups (A-D, according to (Neuzillet et al., 2018)) were cultured and subsequently performed LC-MS/MS based lipid mediator profiling. Heatmap analysis representing each of the four CAF groups along with normal PSCs (PS1) in columns and the identified lipid mediators in rows. Euclidean distance measure was used with Ward clustering algorithm and autoscaling of the mediators in unsupervised clustering (n=1 biological replicate).

Supplementary Figure 4 mRNA expression for critical SPM enzymes in PSC/CAF **(A)** Human PS1 treated over time course to show changes (fold-change) in gene expression for SPM biosynthetic enzymes, including cyclooxygenases and lipoxygenases, after treatment with ATRA. n=2 biological repeats. PSC (blue) and primary CAFs sub-categorized into four groups **(A-D)**, according to Nuezillet 2019 (12) and herein ordered by patient prognosis: C (green), B (yellow), A (brown) and D (cyan), from better to worse prognosis) were either **(B)** cultured to confluency or **(C)** treated with ATRA 1µM daily for 7 days (red) or vehicle control (blue) and subsequently RNA was extracted. qRT-PCR was performed using ALOX12, ALOX15, ALOX15B, COX-1 and COX-2 primers against a GAPDH house-keeping gene. PS1 **(A)** or vehicle control for each subtype **(B)** was used as a reference sample for 2^-ΔΔCt^ formula normalization. Welch’s *t*-test. * *p*<0.05; ** *p*<0.01. n = 3 (biological repeats), **(D)** ALOX5/GAPDH gene specific primer cDNA synthesis followed by real time quantitative PCR (showing delta cycle threshold, delta CT) performed on PSCs (PS1) cells treated with ATRA 1µM for 7 days daily (n=4), TGF-β 5ng/ml for 3 days daily (n=4) or MIAPaCa-2 conditioned media for 7 days (n=2). Student’s *t*-test, * *p*<0.05 against vehicle control. n = 3 (biological repeats).

Supplementary Figure 5 PSC and CAF ALOX15 protein expression. **(A)** PSC (PS1) and **(B-E)** CAFs representative immunocytochemistry images for ALOX15 in ATRA-treated cells (middle row in panel) compared to vehicle controls (top). Respective negative IgG control (bottom) antibodies were used for normalization. ALOX15 is pseudo-colored in red, α-SMA in green, DAPI in blue and merged channels to the right of each row. Scale bar = 100µm. n = 3 (biological repeats).

Supplementary Figure 6 PSC and CAF ALOX12 protein expression and localization. **(A)** PSC (PS1) and **(B-E)** CAFs representative immunocytochemistry images for ALOX12 in ATRA-treated cells (middle row in panel) compared to vehicle controls (top). Respective negative IgG control (bottom) antibodies were used for normalization. ALOX12 is pseudo-colored in red, α-SMA in green, DAPI in blue and merged channels to the right of each row. Scale bar = 100µm. n = 3 (biological repeats).

Supplementary Figure 7 PSC and CAF COX-2 protein expression and localization. **(A)** PSC (PS1) and **(B-E)** CAFs representative immunocytochemistry images for COX2 in ATRA-treated cells (middle row in panel) compared to vehicle controls (top). Respective negative IgG control (bottom) antibodies were used for normalization. COX-2 is pseudo-colored in red, α-SMA in green, DAPI in blue and merged channels to the right of each row. Scale bar = 100µm. n = 3 (biological repeats).

Supplementary Figure 8 mRNA expression for critical SPM receptors in PSC/CAF. PSC (blue) and primary CAFs sub-categorized into four groups (A-D, according to ^82^ and herein ordered by patient prognosis: C (green), B (yellow), A (brown) and D (cyan), from better to worse prognosis) were either **(A)** cultured to confluency or **(B)** treated with ATRA 1µM daily for 7 days (red) or vehicle control (blue) and subsequently RNA was extracted. qRT-PCR was performed using GPR101, GRP18, GPR37, BLT-1, FPR2, ChemR23, GPR32 and LGR6 primers against a GAPDH house-keeping gene. PS1 **(A)** or vehicle control for each subtype **(B)** was used as a reference sample for 2^-ΔΔCt^ formula normalization. Welch’s *t*-test. **p*<0.05; ***p*<0.01; ****p*<0.001; *****p*<0.0001. n = 3 (biological repeats).

Supplementary Figure 9 mRNA expression for critical SPM enzymes and receptors in cancer cells. Real time quantitative PCR performed on PSCs (blue) and PDAC cell lines AsPC-1 and MIAPaCa-2 (yellow). **(A)** SPM biosynthetic enzymes ALOX5, ALOX12, ALOX15, ALOX15B, COX-1 and COX-2 primers and **(B)** SPM receptors ALXR, GRP32, GPR18, GPR37, BLT-1, and ChemR23 primers were assayed against GAPDH as a loading control. Bars represent relative expression compared to house-keeping gene (GAPDH, relative 2^-ΔΔCt^ data) and to vehicle control (normalized to 1) median ± interquartile range (n=3). Welch’s *t*-test. **p*<0.05. n = 3 (biological repeats).

Supplementary Figure 10 Organotypic and spheroid co-culture models and use of PD146176. **(A)** Representative images for each of the conditions representing one 30X H&E image from organotypic cultures model. Organotypic cultures were performed where co-cultures were treated with either vehicle control (blue), PD146176 (5µM, tan), and/or RvD5_n-3 DPA_ (1 or 10nM, 7 days, shades of purple). Scale bar = 300µm **(B)** Relative invaded cell quantifications are performed where each data point reflects one organotypic culture (different shapes represent technical replicates within a biological replicate (n=3)). **(C)** Spheroid cultures were performed where PSCs were pre-treated with either vehicle control (blue), ATRA (1µM, 7 days, pink), PD146176 (5µM, 3 days, tan), and/or RvD5_n-3 DPA_ (1 or 10nM, 7 days, shades of purple) and then co-cultured with cancer cells to form spheres and subsequently embedded in Matrigel/collagen gels and allowed to invade for 2 days. Representative bright-field 20x microscopy images Scale bar = 200µm. **(D)** Relative invaded cell quantifications are performed where each data point reflects one sphere. Experiments were performed three independent times (different shapes, multiple technical and biological replicates). Kruskal-Wallis with Dunn’s multiple comparisons test. **p*<0.05, ***p*<0.01, ****p*<0.001, *****p*<0.0001. **(E)** Representative 30X H&E field images for cancer cell alone organotypic assays (no PSCs, MiaPaCa2 and AsPC1) revealing no invasion. Scale bar = 300µm.

Supplementary Figure 11 Compound cell viability assay and ShRNA ALOX15. **(A)** MTS 72h viability assay for PD146176, RvD5_n-3 DPA_ and ATRA in PS1 and MIAPaCa-2 cells. Cells were cultured at 2x10^6^ for MIAPaCa-2 or 1x10^6^ for PS1, treated with the drug for 72h and viability assessed by MTS reagent incubation after 1h and absorbance measured at 492nm. Results represent relative absorbance to vehicle control. n=3-5 (biological repeats). One-way ANOVA. **(B)** qRT-PCR (n=3) performed on cultured PS1 cells transfected with lentiviral particles containing ALOX15 or scrambled (SCR) shRNA plasmid confirming ALOX15 RNA knock down. Welch’s *t*-test. **p*<0.05. n = 3 (biological repeats).

Supplementary Figure 12 Principal component analysis of SPM production in human plasma. LC-MS/MS lipid mediator profiling using plasma from a cohort of 20 PDAC patients (red) and 22 age- and gender-matched healthy volunteers (green). Multivariate PCA scores plot analyses were carried out to understand clustering and differentiation according to lipid mediators of **(A)** PDAC (n=20) vs healthy volunteers (n=22), **(B)** non-metastatic PDAC (stages I-III, n=11) vs metastatic PDAC (stage IV, n=9) vs healthy volunteers (n=22), and **(C)** non-metastatic (n=11) vs healthy volunteers (n=22).

Supplementary Figure 13 Cell Profiler subcellular intensity measuring pipeline. **(A)** Image selection for pipeline input. **(B)** Median filter applied to individual channels to reduce salt-and-pepper noise while preserving borders. A reduction of noise was performed in the DAPI channel, which performs non-local means noise reduction. Instead of only using a neighborhood of pixels around a central pixel for de-noising, such as in Gaussian filter, multiple neighborhoods are pooled together. The neighborhood pool is determined by scanning the image for regions similar to the area around the central pixel using a correlation metric and a cut-off value. This helped to smooth DAPI-stained nuclei for more precise border detection. **(C)** Then an identification of primary object was performed using the DAPI channel to create nuclei masks. From the nuclei masks, an expansion of 1µm was performed on the edge of the nucleus. Then a shrinkage of the nucleus mask of 1µm was carried on. Both created two separate masks. A secondary object was created using α-SMA staining at 488nm as a cell shape marker. The software identifies the cells by using the nuclei as a “seed” region, then growing outwards until stopped by the image threshold or by a neighbor. A global minimum cross entropy algorithm thresholding strategy was used as a propagation method to delineate the boundary between neighboring cells. **(D)** Tertiary objects (masks) were created by defining regions: cytoplasm, nuclear and perinuclear space. Then pixel intensity within the regions was measured along with other pattern measurements in the 594nm channel for the protein of interest.

Supplementary Table 1 PSC (PS1) Lipid Mediator Profiling. PS1 cell cultures treated with ATRA, TGF-β or vehicle control (EtOH) lipid mediator concentrations (n=3 biological repeats). (-) below limits of detection; all mediators not shown also under detection limits.

Supplementary Table 2 Summary of MS/MS transitions for lipid mediator profiling.

Supplementary Table 3 Summary of antibodies used for immunocytochemistry.
