# Corrigendum: Arachidonate 15-lipoxygenase-mediated production of Resolvin D5_n-3 DPA_ abrogates pancreatic stellate cell-induced cancer cell invasion

**DOI:** 10.3389/fimmu.2024.1353835

**Published:** 2024-01-24

**Authors:** Gabriel A. Aguirre, Michelle R. Goulart, Barts Pancreas Tissue Bank, Jesmond Dalli, Hemant M. Kocher

**Affiliations:** ^1^Centre for Tumour Biology, Barts Cancer Institute, London, United Kingdom; ^2^William Harvey Research Institute, Barts and The London School of Medicine and Dentistry, John Vane Science Centre, Queen Mary University of London, London, United Kingdom

**Keywords:** pancreatic ductal adenocarcinoma, specialized pro-resolving mediator, lipid mediator, cancer-associated fibroblast, all-*trans* retinoic acid, ALOX15, CAF subtypes

In the published article, there was an error in the Supplementary Material: Supplementary Methods, Page 7, at the sentence: “Identification was conducted using published criteria, briefly a minimum of six diagnostic ions in total ion count EPI spectrum, matching retention time to synthetic or authentic standards, MRM peaks with more than five data points, a signal-to-noise ratio > 3 (**Supplementary Figure 2**). [41]. The correct material statement appears below.

“Identification was conducted using published criteria, matching retention time to synthetic or authentic standards, MRM peaks with  more than five data points, a signal-to-noise ratio > 3 (UK/MAP). In a subset of samples the identity of these mediators was further confirmed by matching a minimum of six diagnostic ions in total ion count EPI spectrum to that of a reference standard (**Supplementary Figure 2**). [41]”

The authors apologize for this error and state that this does not change the scientific conclusions of the article in any way. The original article has been updated.

