# Supplementary material for: Arachidonate 15-lipoxygenase-mediated production of Resolvin D5n-3 DPA abrogates pancreatic stellate cell-induced cancer cell invasion
Source: Front Immunol. 2023 Nov 16;14:1248547. doi: 10.3389/fimmu.2023.1248547 (PMC10687150; doi:10.3389/fimmu.2023.1248547)
Supplement: Supplementary Figure 1 — PSC phenotype characterization and quiescence. Representative immunocytochemistry images of fixed PS1 cells on coverslips stained for (A) vimentin, (B) GFAP, (C) Desmin and (D) α-SMA all shown in green after vehicle control (Ctrl, ethanol, 0.1%) or ATRA (1µM) treatment. (E-H) Quantification of mean pixel intensity per coverslip from independent biological replicates (n=3, technical replicates represented by different shapes). Each data-point represents median value from same coverslip of 5-10 cells per coverslip for ATRA (right column, red) and vehicle control (left column, blue) with summary data as median and interquartile range. Mann-Whitney U test. ***p<0.001, ns = not significant. Scale bar: 100µm. Representative Western blots for PSC markers (I) vimentin, (J) GFAP, (K) Desmin and (L) α-SMA, after ethanol (vehicle control, Ctrl) or ATRA treatment along with quantification (M-P) for each marker normalized to HSC70 and to vehicle control from three independent experiments (each dot) with summary data as mean +/- SD. [file DataSheet_2.pdf]

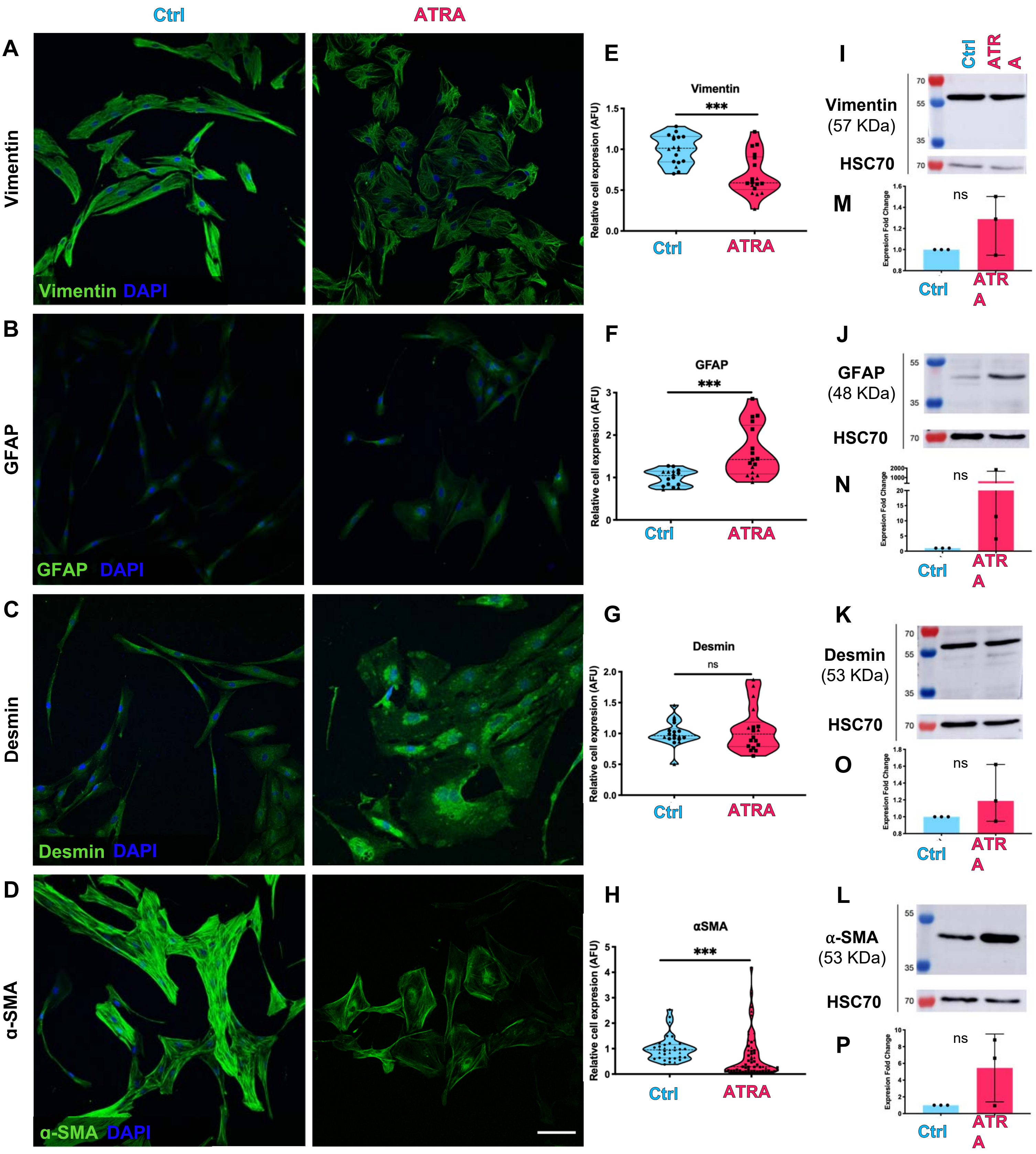

Supplementary Figure 1

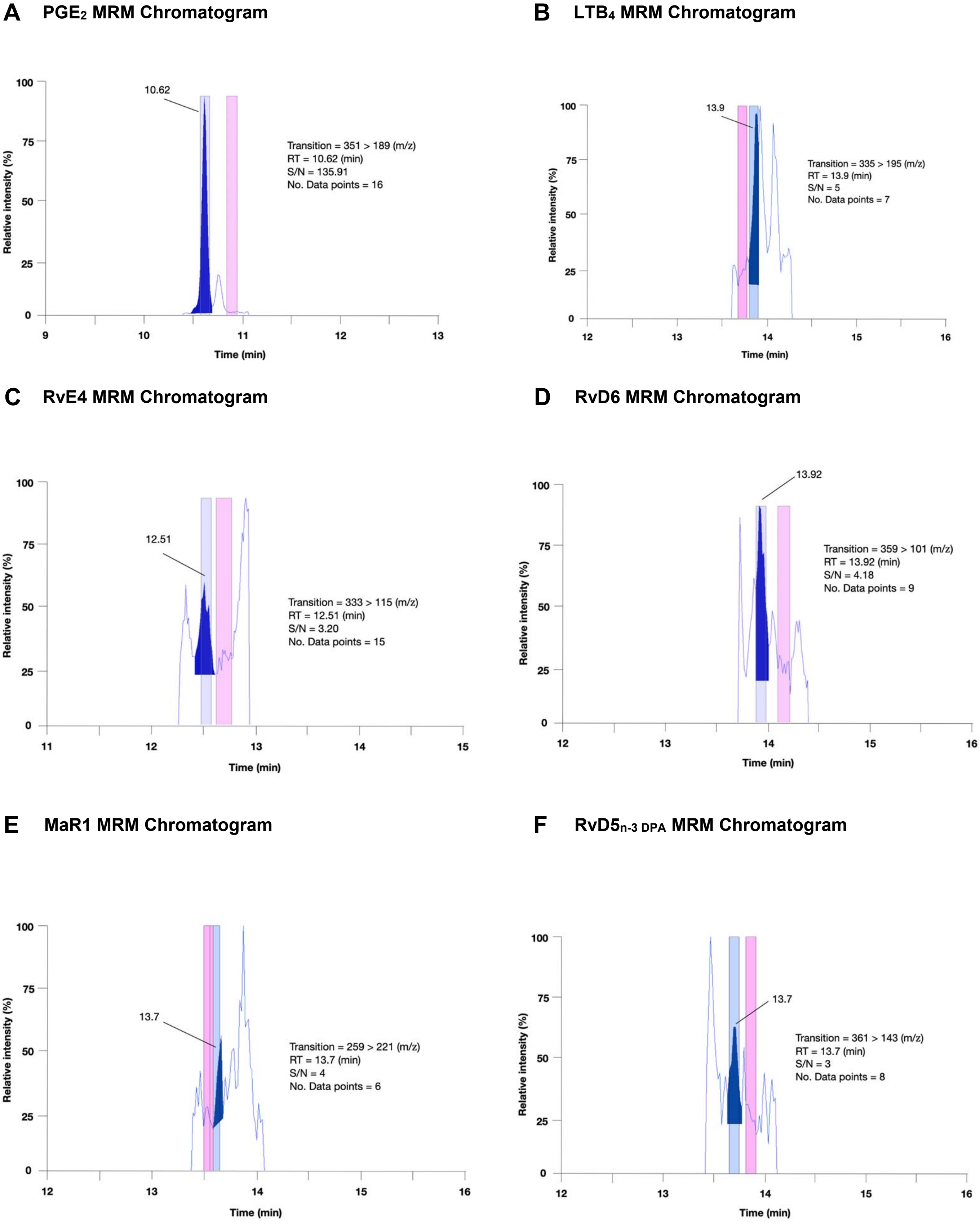

Supplementary Figure 2

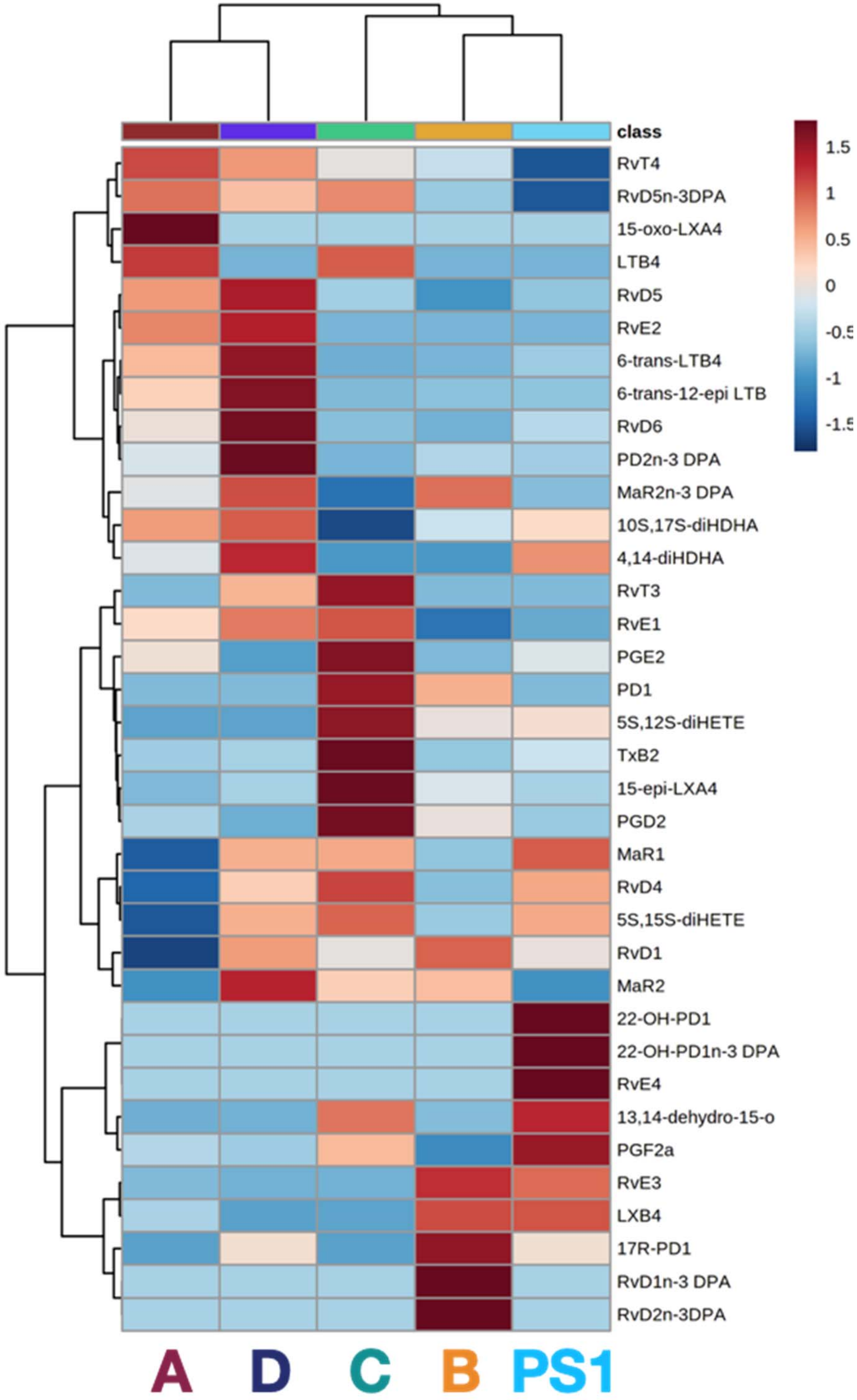

Supplementary Figure 3

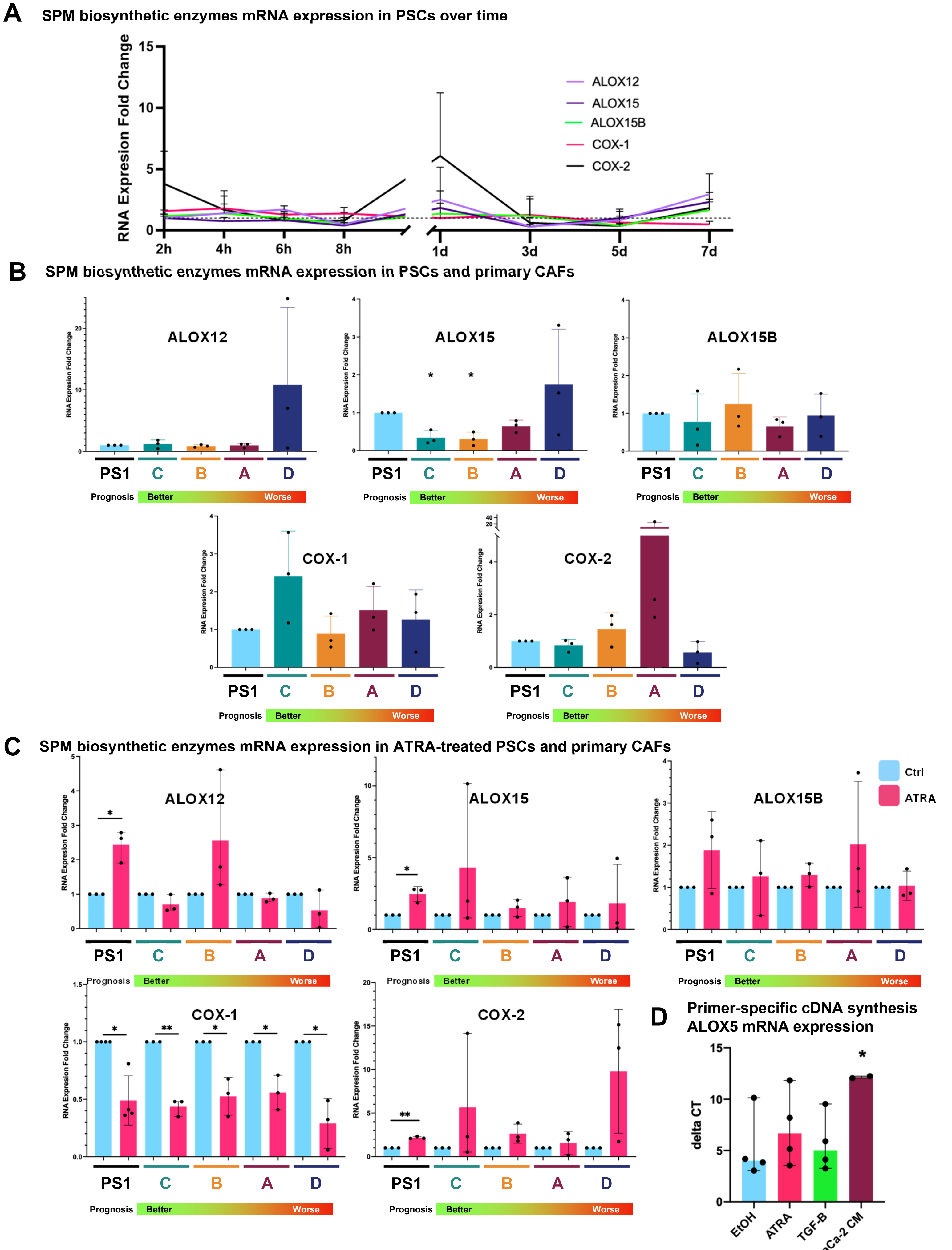

Supplementary Figure 4

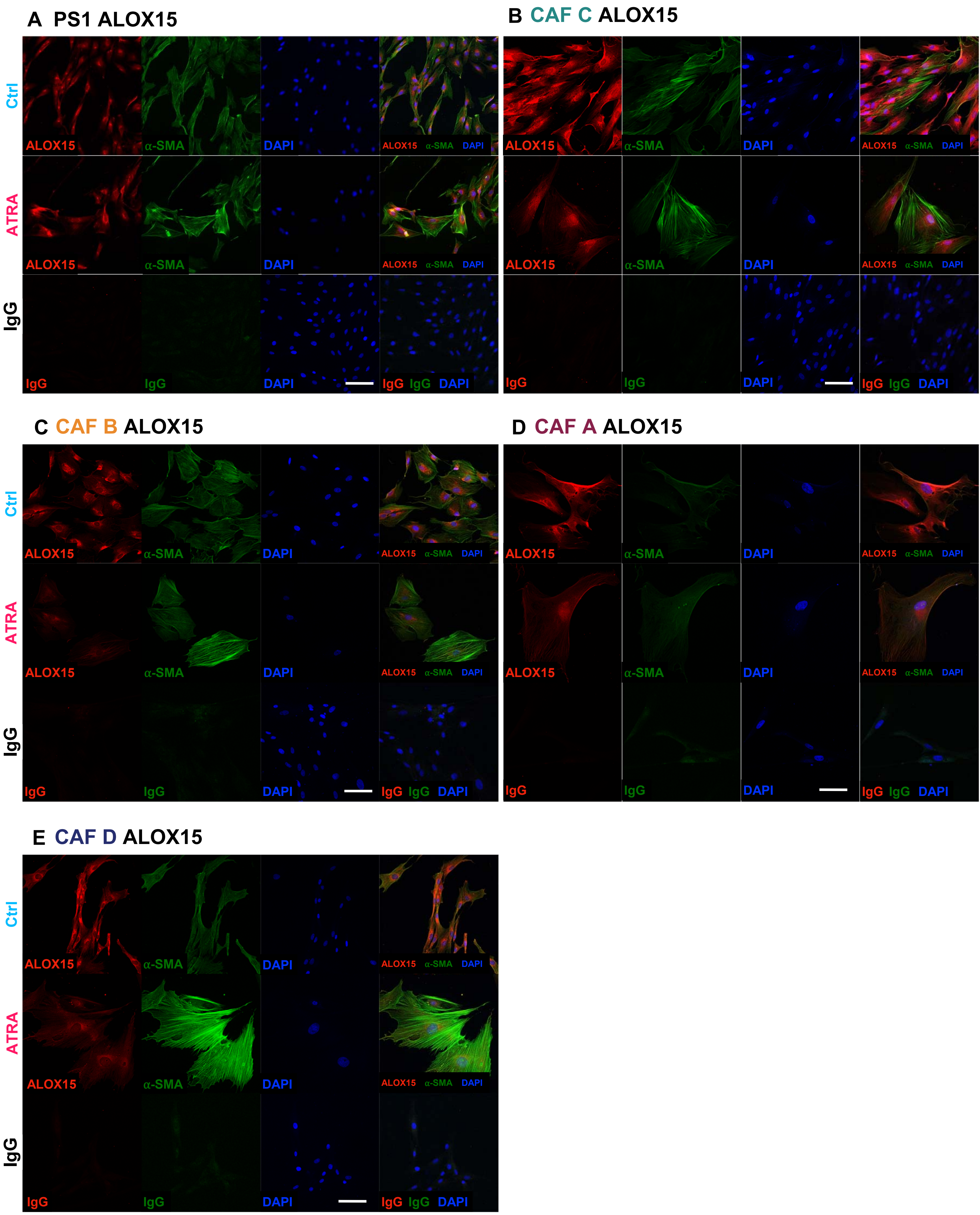

Supplementary Figure 5

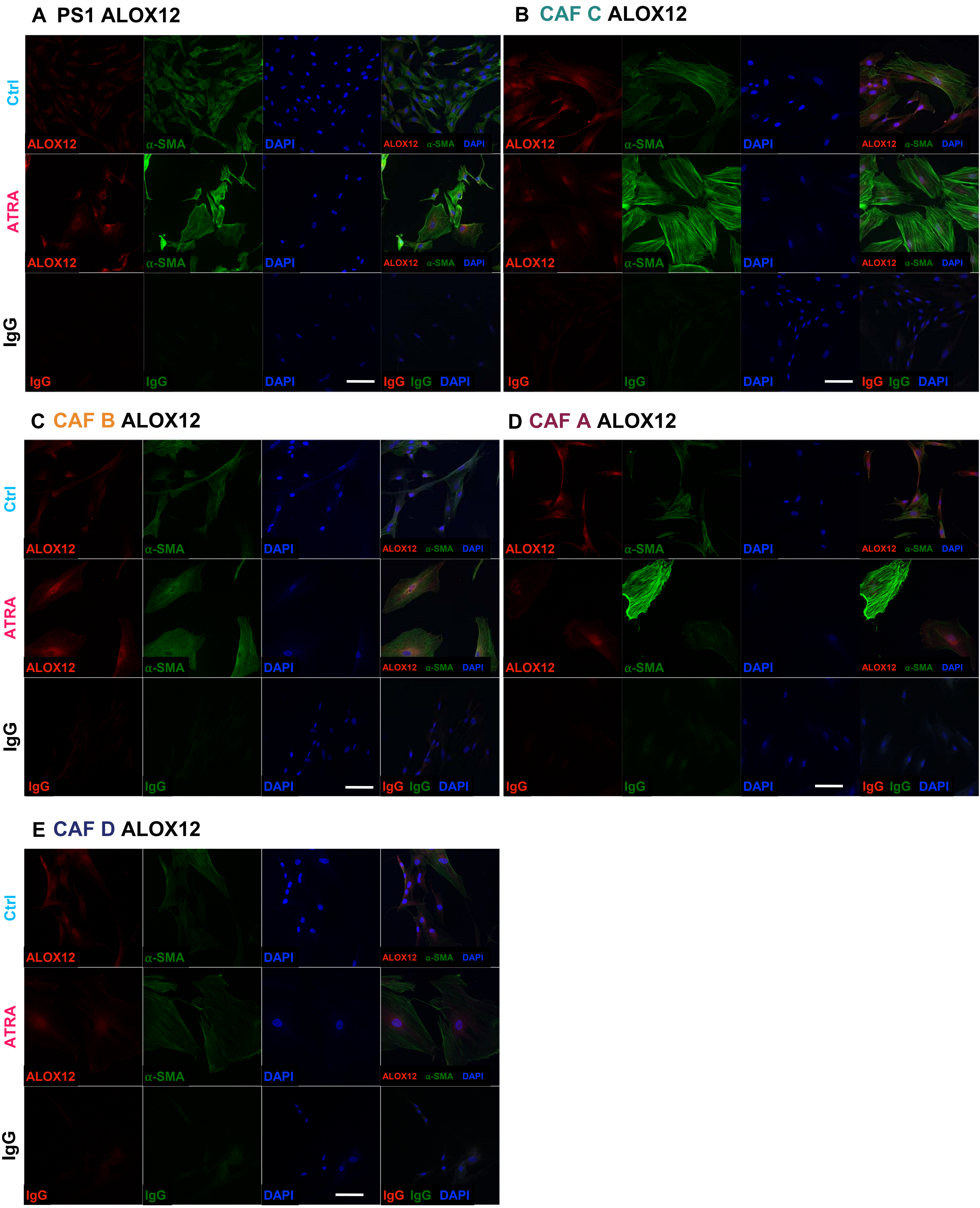

Supplementary Figure 6

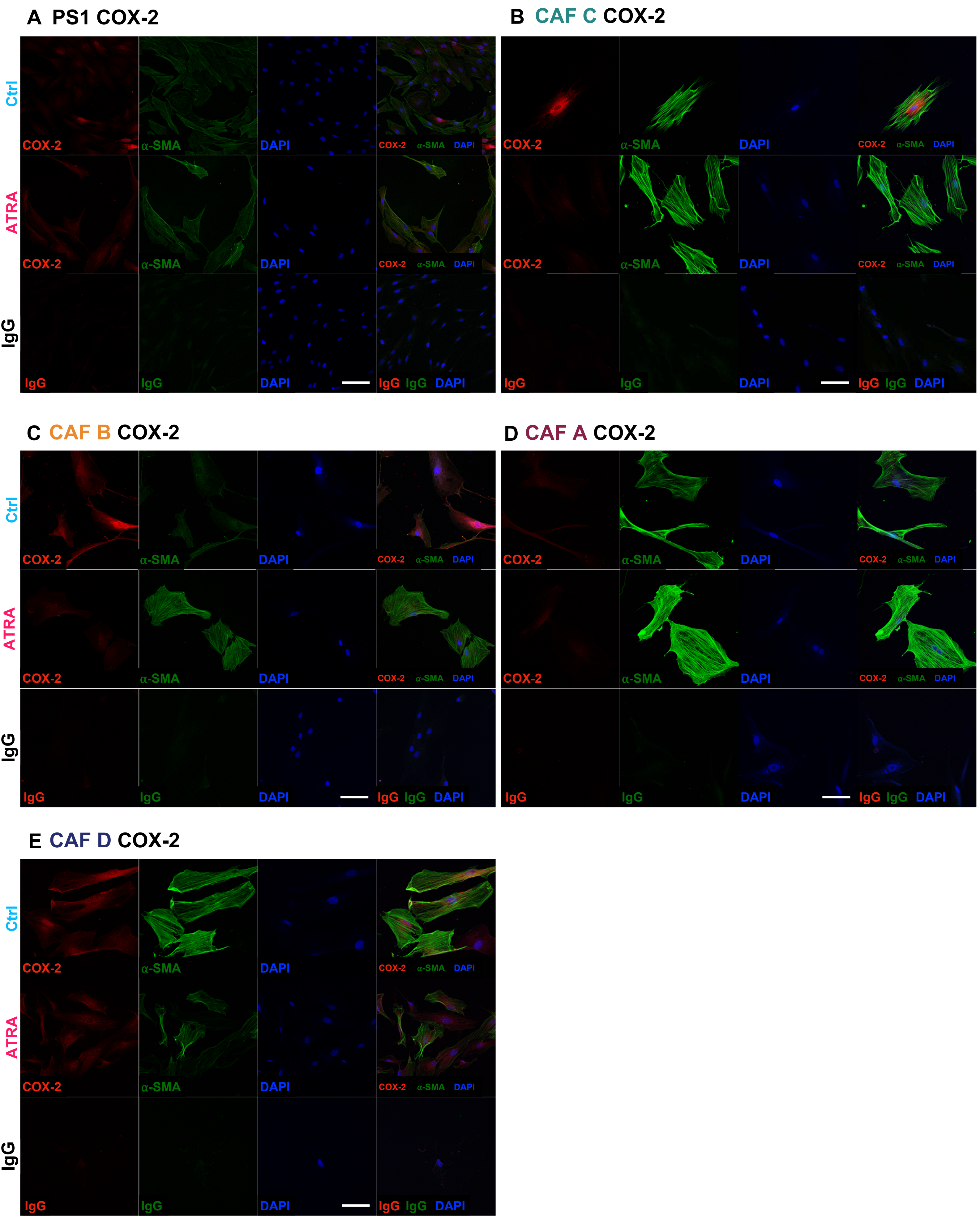

Supplementary Figure 7

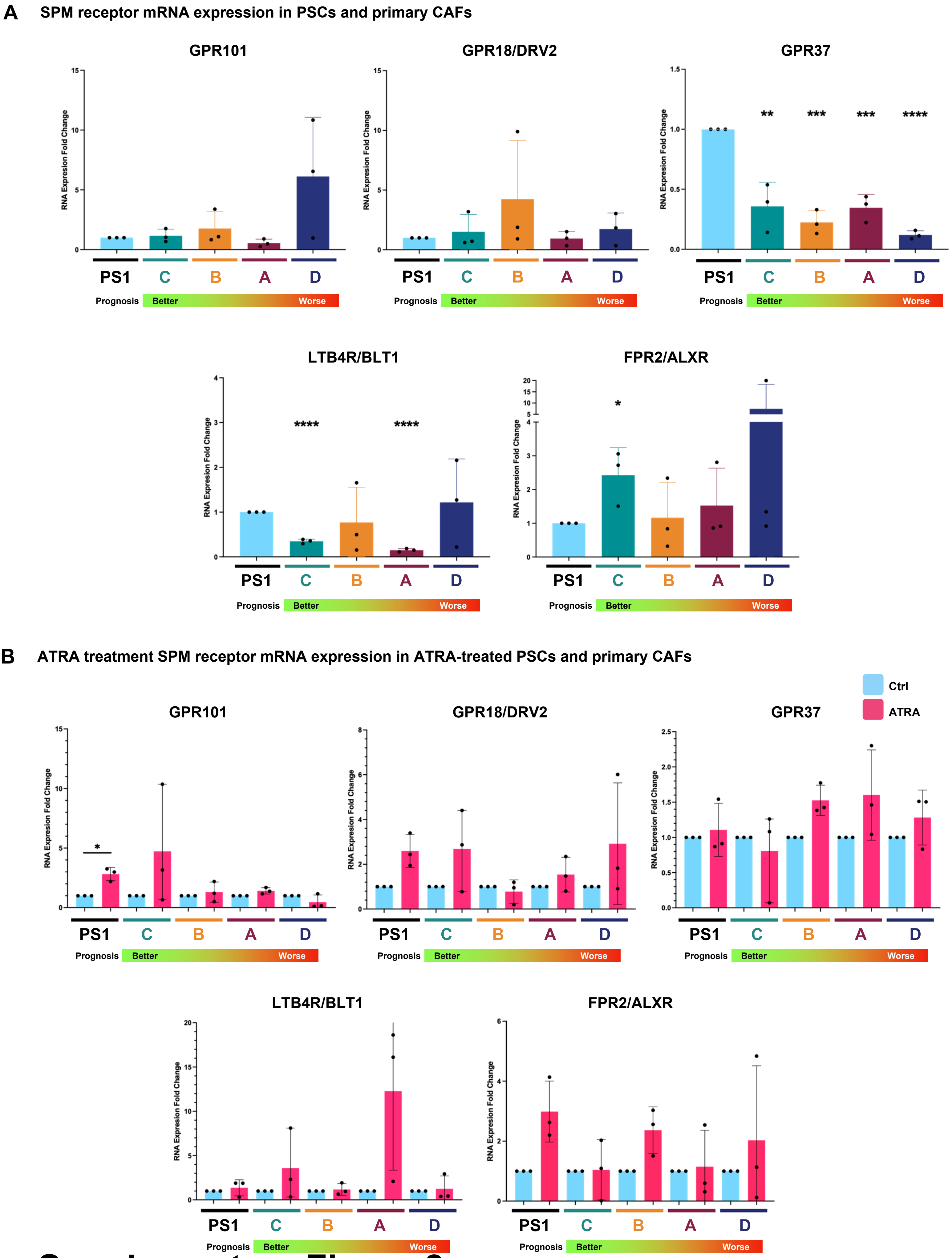

Supplementary Figure 8

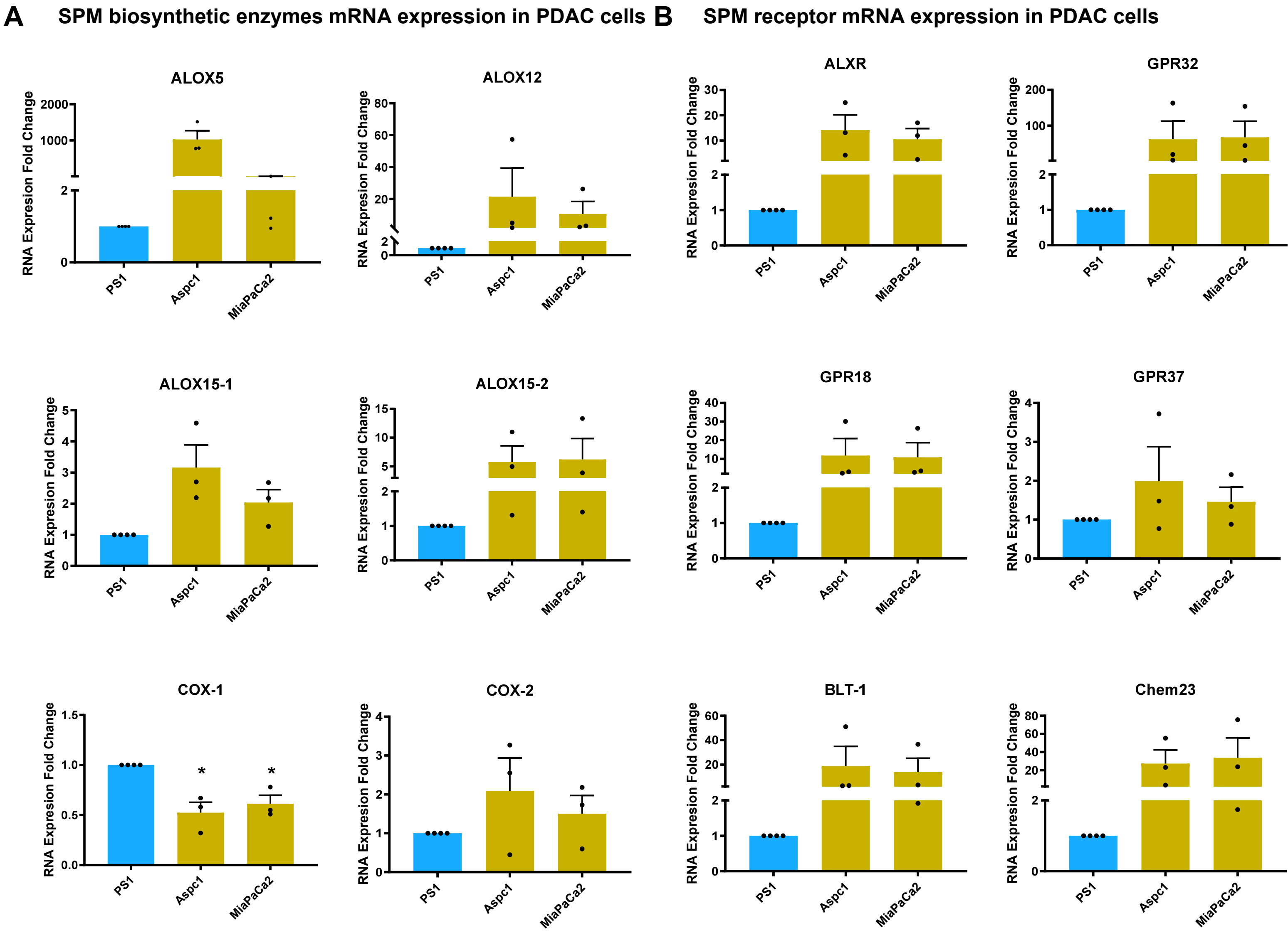

Supplementary Figure 9

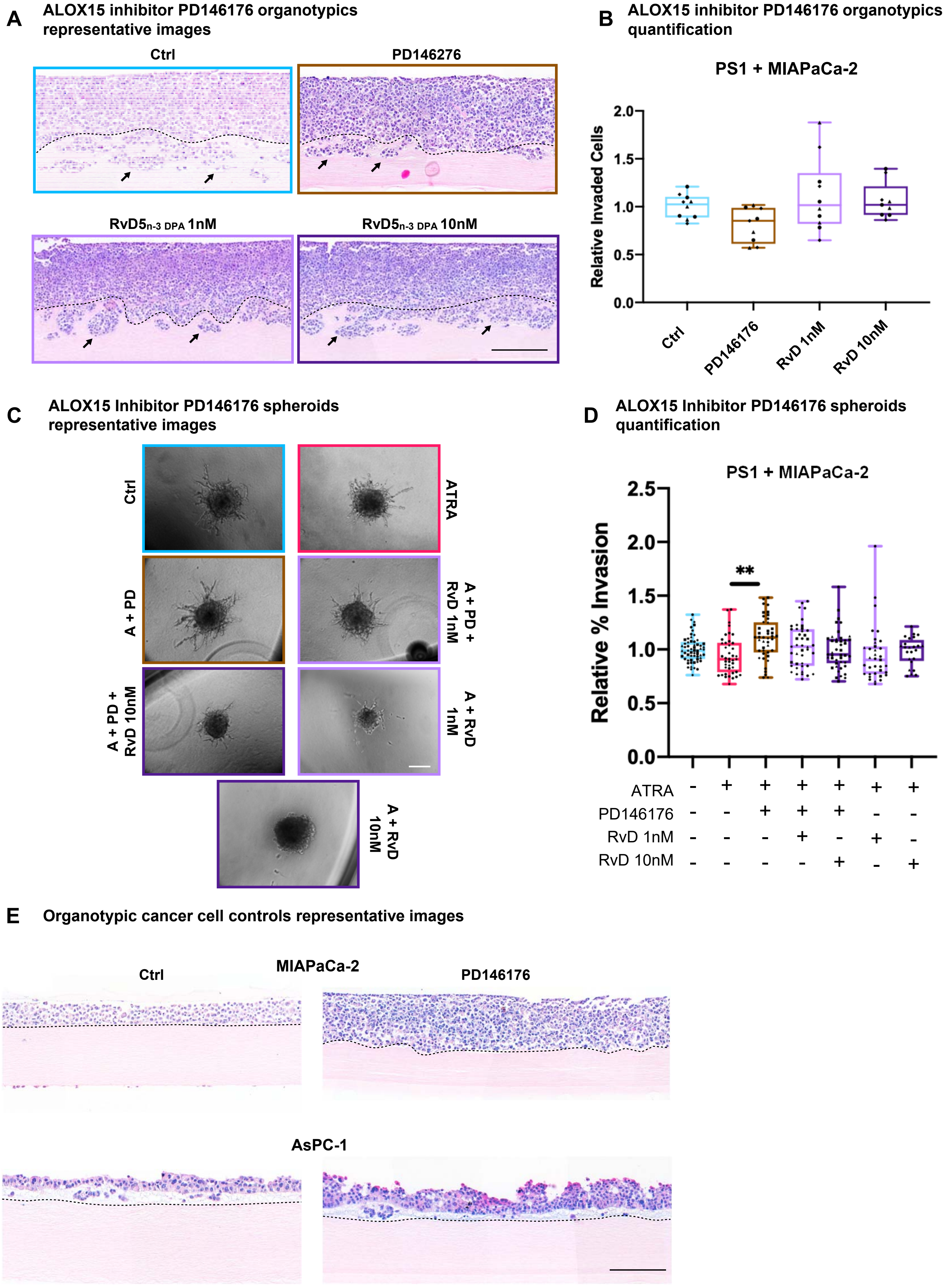

Supplementary Figure 10

A MTS cell viability assay at 72h

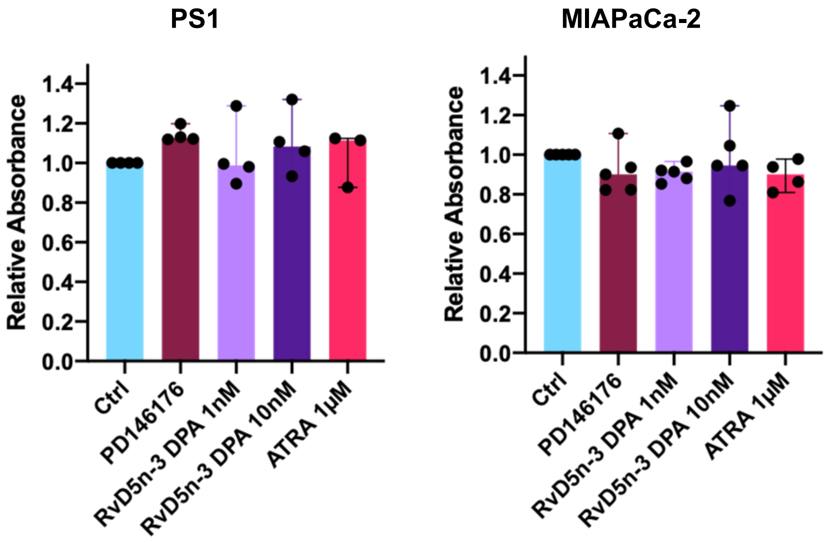

B ALOX 15 shRNA knock down qPCR confimation

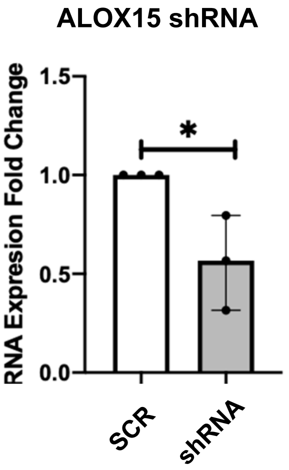

Supplementary Figure 11

A **Healthy vs PDAC PCA**

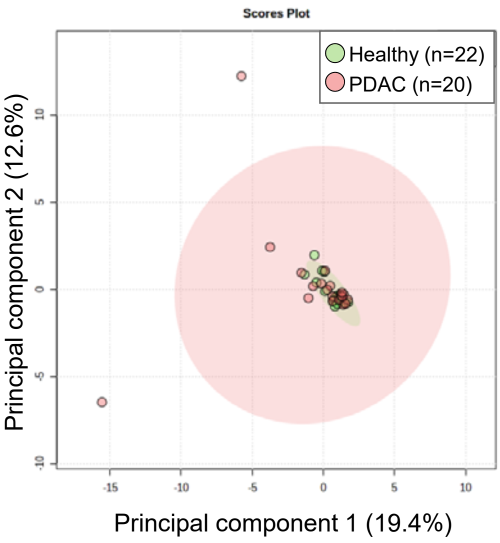

B **Healthy vs PDAC SI-III vs SIV PCA**

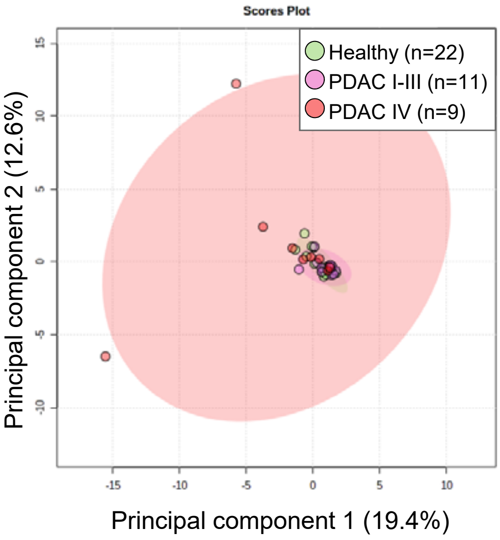

C **Healthy vs PDAC SI-III PCA**

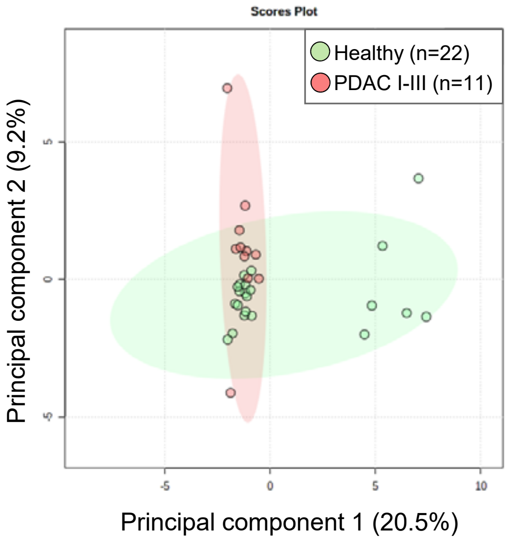

Supplementary Figure 12

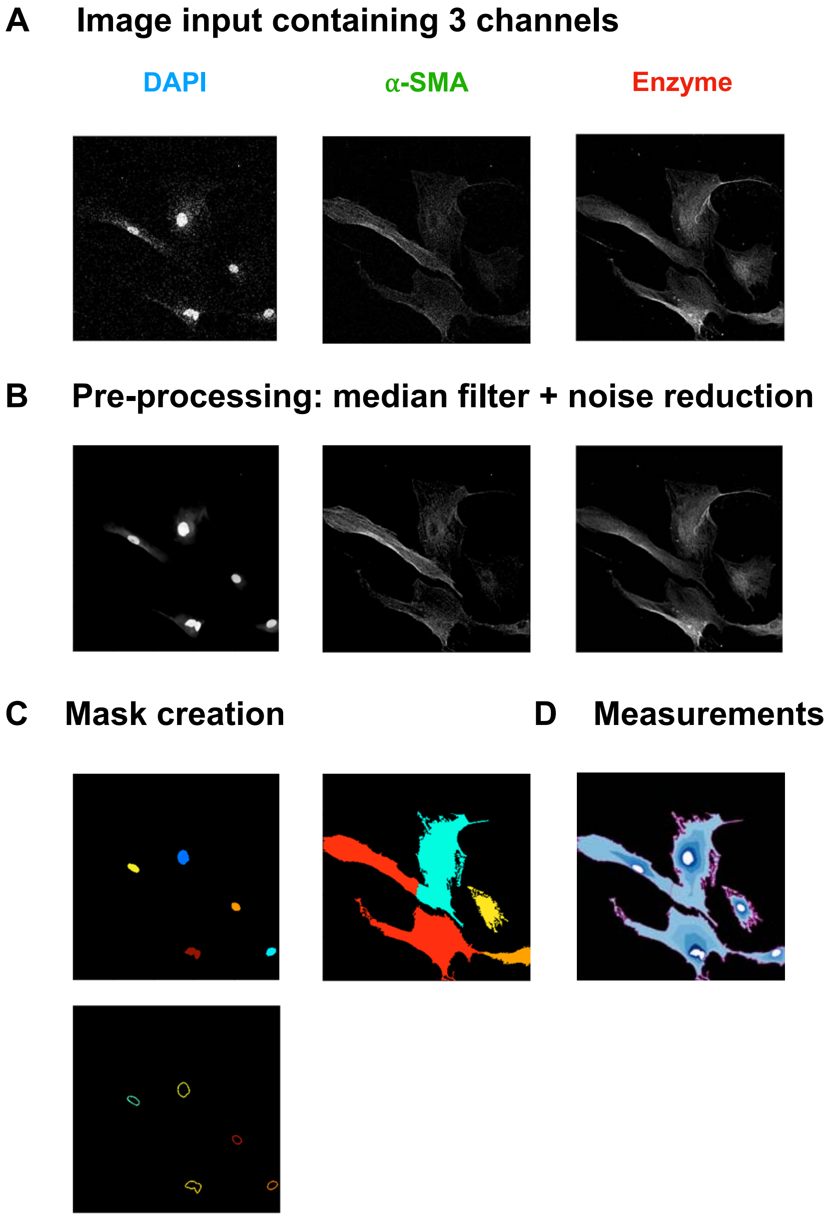

Supplementary Figure 13
