# Supplementary material for: Arachidonate 15-lipoxygenase-mediated production of Resolvin D5n-3 DPA abrogates pancreatic stellate cell-induced cancer cell invasion
Source: Front Immunol. 2023 Nov 16;14:1248547. doi: 10.3389/fimmu.2023.1248547 (PMC10687150; doi:10.3389/fimmu.2023.1248547)
Supplement: Supplementary Table 1 — PSC (PS1) Lipid Mediator Profiling. PS1 cell cultures treated with ATRA, TGF-β or vehicle control (EtOH) lipid mediator concentrations (n=3 biological repeats). (-) below limits of detection; all mediators not shown also under detection limits. [file DataSheet_1.doc]

**Arachidonate 15-lipoxygenase mediated production of Resolvin D5_n-3 DPA_ abrogates pancreatic stellate cell induced cancer cell invasion.**

*Gabriel A Aguirre^1^, Michelle R Goulart^1^, Barts Pancreas Tissue Bank^1^, Jesmond Dalli ^2*^, Hemant M Kocher^1*^.*

^1^ Centre for Tumour Biology, Barts Cancer Institute, ^2^ William Harvey Research Institute, Barts and The London School of Medicine and Dentistry, Queen Mary University of London, John Vane Science Centre, Charterhouse Square, London, EC1M 6BQ. England.

## Supplementary Tables

**Supplementary Table 1.** **PSC (PS1) Lipid Mediator Profiling.** *PS1 cell cultures treated with ATRA,* TGF-β *or vehicle control (EtOH) lipid mediator concentrations (n=3 biological repeats). (-) below limits of detection; all mediators not shown also under detection limits.*

|  |  | **Mediator (𝘱g/ml)** | **EtOH1** | **EtOH2** | **EtOH3** | **ATRA1** | **ATRA2** | **ATRA3** | **TGFβ1** | **TGFβ2** | **TGFβ3** |
| --- | --- | --- | --- | --- | --- | --- | --- | --- | --- | --- | --- |
| **DHA** | **RvD** | **RvD4** | 0.20 | 0.13 | 0.25 | 0.44 | 0.38 | 0.37 | 0.43 | 0.54 | 0.32 |
|  |  | **RvD5** | 0.01 | - | - | - | - | - | - | - | - |
|  |  | **RvD6** | 0.07 | 0.06 | 0.09 | 0.44 | 0.61 | 0.68 | 0.29 | 0.19 | 0.22 |
|  | **MaR** | **MaR1** | 0.12 | 0.08 | 0.15 | 0.56 | 0.59 | 0.82 | - | - | - |
|  |  | **7S,14S-diHDHA** | 0.18 | - | 0.26 | 0.97 | 0.90 | 0.63 | - | - | - |
|  |  | **4,14-diHDHA** | - | 0.01 | 0.01 | 0.10 | 0.14 | 0.13 | 0.04 | 0.05 | - |
| **n-3 DPA** | **RvT** | **RvT4** | 0.02 | 0.02 | - | 0.14 | 0.11 | 0.11 | - | 0.07 | 0.03 |
|  | **RvD_n-3 DPA_** | **RvD1_n-3 DPA_** | - | 0.02 | 0.04 | 0.12 | 0.09 | 0.13 | 0.10 | 0.11 | 0.08 |
|  |  | **RvD2_n-3 DPA_** | - | 0.03 | 0.06 | - | - | - | - | - | - |
|  |  | **RvD5_n-3 DPA_** | - | - | - | 0.15 | 0.16 | 0.12 | 0.09 | - | 0.05 |
| **EPA** | **RvE** | **RvE1** | - | - | - | - | - | - | 0.40 | 0.63 | 0.30 |
|  |  | **RvE2** | 0.01 | 0.10 | 0.21 | 0.39 | 0.47 | 0.45 | 0.22 | 0.27 | 0.23 |
|  |  | **RvE3** | 0.18 | 0.11 | 0.16 | - | - | - | 0.37 | 0.58 | 0.29 |
|  |  | **RvE4** | - | 0.02 | 0.06 | - | - | - | 0.17 | 0.18 | 0.11 |
| **AA** | **LX** | **LXB4** | 4.26 | 2.34 | 3.74 | 9.84 | 5.94 | - | 6.50 | 9.52 | 4.93 |
|  |  | **5S,15S-diHETE** | 0.79 | 0.64 | 1.14 | 4.61 | 4.74 | 4.95 | 2.25 | 3.05 | 2.17 |
|  |  | **13,14-dehydro-15-oxo-LXA5** | 6.18 | 3.38 | 5.54 | 16.00 | 13.91 | 14.91 | 10.68 | 12.40 | 7.27 |
|  |  | **15-epi-LXA4** | 0.24 | 0.14 | 0.27 | 0.79 | 0.74 | 1.02 | 0.50 | 0.63 | 0.32 |
|  | **LT** | **LTB_4_** | 0.05 | 0.03 | 0.04 | - | - | - | 0.10 | 0.15 | 0.12 |
|  |  | **5S,12S-diHETE** | 0.05 | 0.02 | - | - | 0.22 | - | 0.15 | 0.11 | 0.07 |
|  |  | **6-trans-LTB4** | 0.04 | 0.02 | 0.03 | 0.16 | 0.16 | 0.20 | 0.08 | 0.10 | 0.05 |
|  |  | **6-trans-12-epi LTB4** | 0.04 | 0.02 | 0.03 | 0.17 | 0.13 | 0.17 | 0.08 | 0.11 | 0.05 |
|  | **PG** | **PGE_2_** | 3.33 | 3.06 | 2.85 | 54.70 | 64.01 | 70.99 | 9.92 | 8.51 | 8.65 |
|  |  | **PGD_2_** | 3.26 | 1.74 | 3.67 | 12.02 | 12.53 | 18.23 | 6.03 | 7.21 | 5.87 |
|  |  | **PGF_2a_** | 3.43 | 6.73 | 8.24 | 23.55 | 29.72 | 50.28 | 11.06 | 12.16 | 8.08 |
|  | **TX** | **TxB2** | 1.38 | 0.80 | 1.63 | 5.92 | 5.29 | 7.07 | 4.43 | 4.84 | 3.21 |

**Supplementary Table 2. Summary of MS/MS transitions for lipid mediator profiling.**

| **Mediator** | **Transition** | | **Mediator** | **Transition** | |
| --- | --- | --- | --- | --- | --- |
| **DHA Bioactive Metabolome** | **Q1** | **Q3** | **n-3 DPA bioactive Metabolome** | **Q1** | **Q3** |
| RvD1 | ***375*** | ***215/233*** | RvT1 | ***377*** | ***221/193*** |
| RvD2 | ***375*** | ***215/141*** | RvT2 | ***377*** | ***197*** |
| RvD3 | ***375*** | ***147/137*** | RvT3 | ***377*** | ***197/173*** |
| RvD4 | ***375*** | ***101/225*** | RvT4 | ***361*** | ***211/193*** |
| RvD5 | ***359*** | ***199/141*** |  |  |  |
| RvD6 | ***359*** | ***159/101*** | RvD1_n-3 DPA_ | ***377*** | ***143/215*** |
| 17R-RvD1 | ***375*** | ***215/233*** | RvD2_n-3DPA_ | ***377*** | ***233/261*** |
| 17R-RvD3 | ***375*** | ***147/137*** | RvD5_n-3DPA_ | ***361*** | ***199/143/263*** |
|  |  |  |  |  |  |
| PD1 | ***359*** | ***153/137*** | PD1_n-3 DPA_ | ***361*** | ***183/155*** |
| 17R-PD1 | ***359*** | ***153/137*** | PD2_n-3 DPA_ | ***361*** | ***233*** |
| 10S,17S-diHDHA | ***359*** | ***137/153*** | 10S, 17S-diHDPA | ***361*** | ***183/155*** |
| 22-OH-PD1 | ***375*** | ***153/137*** | 22-OH-PD1_n-3 DPA_ | ***377*** | ***183*** |
|  |  |  |  |  |  |
| MaR1 | ***359*** | ***221/177*** | MaR1_n-3 DPA_ | ***361*** | ***223*** |
| MaR2 | ***359*** | ***191/221*** | MaR2_n-3 DPA_ | ***361*** | ***193/223*** |
| 22-OH-MaR1 | ***375*** | ***221/177*** | 7S,14S-diHDPA | ***361*** | ***223*** |
| 22-COOH-MaR1 | ***221*** | ***221*** |  |  |  |
| 14-oxo-MaR1 | ***357*** | ***248*** | **AA bioactive Metabolome** |  |  |
| 7S,14S-diHDHA | ***359*** | ***177/221*** | LXA_4_ | ***351*** | ***115/217*** |
| 4S,14S-diHDHA | ***359*** | ***159/101*** | LXB_4_ | ***351*** | ***221/115*** |
|  |  |  | 5S,15S-diHETE | ***335*** | ***115/235*** |
| **EPA bioactive Metabolome** |  |  | 15-epi-LXA_4_ | ***351*** | ***217/115*** |
| RvE1 | ***349*** | ***161/195*** | 15-epi-LXB_4_ | ***351*** | ***115/221*** |
| RvE2 | ***333*** | ***159/199*** | 13,14-dehydro-15-oxo-LXA_4_ | ***351*** | ***215/115*** |
| RvE3 | ***333*** | ***201/275*** | 15-oxo-LXA_4_ | ***349*** | ***115*** |
| RvE4 | ***333*** | ***115*** |  |  |  |
|  |  |  | LTB_4_ | ***335*** | ***195*** |
| **Deuterated Standards** |  |  | 5S,12S-diHETE | ***335*** | ***195*** |
| d8-5HETE | ***327*** | ***116*** | 6-trans-LTB_4_ | ***335*** | ***195*** |
| d_4_-LTB_4_ | ***339*** | ***197*** | 6-trans-12-epi LTB_4_ | ***335*** | ***195*** |
| d_5_-MaR1 | ***364*** | ***177*** | 20-OH-LTB_4_ | ***351*** | ***195*** |
| d_5_-MaR2 | ***364*** | ***177*** | 20-COOH-LTB_4_ | ***365*** | ***195*** |
| d_4_-PGE_2_ | ***355*** | ***275*** |  |  |  |
| d_5_-LXA_4_ | ***356*** | ***115*** | PGD_2_ | ***351*** | ***189*** |
| d_5_-RvD3 | ***380*** | ***147*** | PGE_2_ | ***351*** | ***189*** |
| d_5_-RvD2 | ***380*** | ***141*** | PGF_2a_ | ***353*** | ***193*** |
| d_4_-RvE1 | ***353*** | ***197*** | TxB_2_ | ***369*** | ***169*** |
| d_5_-17RRvD1 | ***380*** | ***141*** |  |  |  |

**Supplementary Table 3. Summary of antibodies used for immunocytochemistry.**

| Antibody | Host species | Target species | Supplier  (Cat. No.) | Clone | Dilution |
| --- | --- | --- | --- | --- | --- |
| ⍺SMA | Mouse | Human | Dako (M0851) | 1A4 | 1:200 |
| Desmin | Mouse | Human | Sigma (D1033) | DE-U-10 ascites | 1:100 |
| GFAP | Mouse | Human | Sigma (G3893) | G-A-5 ascites | 1:50 |
| Vimentin | Mouse | Human | Dako (M0725) | V9 | 1:500 |
| ALOX5 | Rabbit | Human | Sigma/Atlas (HPA071285) |  | 1:50 |
| ALOX12 | Rabbit | Human | Sigma/Atlas (HPA010691) |  | 1:100 |
| ALOX15-1 | Rabbit | Human | Sigma/Atlas (HPA013859) |  | 1:100 |
| ALOX15-2 | Rabbit | Human | Sigma/Atlas (HPA010562) |  | 1:50 |
| COX-1 | Rabbit | Human | Sigma/Atlas (HPA002834) |  | 1:100 |
| COX-2 | Rabbit | Human | Sigma/Atlas (HPA001335) |  | 1:50 |
| Alexa Fluor 546 | Goat | Rabbit | Invitrogen (A110771) |  |  |
| Alexa Fluor 488 | Goat | Rabbit | Invitrogen (A110770) |  |  |
| Negative Control IgG | Mouse |  | Dako (X0931) |  |  |
| Negative Control IgG | Rabbit |  | Abcam (ab172730) |  |  |

## Supplementary Methods

### Organotypic Culture Model

Gels were harvested on day 11, fixed in 10% saline buffered formalin (BAF-0010-25A, Cellstor), transferred to 70% ethanol, embedded in paraffin, and cut into 4µm sections. Quantified by summating the number of invaded cells across the gel in 5 serial 30X fields (limiting to within the area of cellularity to avoid edge artefacts). [39]

### Hanging droplet spheroid model

PS1 (4.4 x 104 cells/ml) and MIAPaCa-2 cancer cells (2.2x10^4^ cells/ml) were combined in a 0.24% methylcellulose solution (M0512, Sigma-Aldrich). Droplets containing 1000 cells were then plated on the underside of a 15 cm culture dish and left to form spheroids overnight at 37ºC. Spheroids were then collected and centrifuged at 100x g for 4 minutes and washed twice with normal media. Cells were then resuspended in gel composed of 1.6 mg/ ml Collagen I (internally prepared from rat rail) and 17.5% Matrigel (354234, Corning), prepared in PS1 culture medium and buffered to physiological pH with NaOH. Approximately 6 spheroids suspended in gel mix were added to a pre-coated well of a low attachment plate and left to solidify at 37ºC before PS1 culture medium was added on top. Spheroids were thereafter incubated for 2 days, and images were taken by light microscopy. Percentage invasion was analyzed using ImageJ and calculated as a measure of the total invasive area relative to the central sphere.

### Lipid Mediator Profiling by Coupled liquid chromatography with tandem mass spectrometry (LC-MS/MS)

The same FBS batch was used throughout the experiment to avoid batch-to-batch differences in precursors present in the serum. On the last day of treatment, DMEM/F12 phenol red-free media (21041025, Gibco) was used to avoid phenol red contamination of the instrument, as it is used at high concentrations. Also, on the last day of treatment, media FBS supplementation was lowered from 10% to 2% to avoid precursor and mediators present in the media interacting with the endogenous cellular biosynthetic capability. Cells were not completely serum-starved (2%) to exclude cellular stress that could potentially alter their metabolism. From each cell culture flask/plate, the media was transferred to a centrifuge tube containing one volume (5ml) of methanol with deuterated internal standards. Cell culture flasks/plates were then washed once with PBS to eliminate any traces of phenol red. Following this, another media volume of methanol plus internal standards was added to the plate well to allow for cell lysis and protein precipitation, making up a total of twice the media volume (10ml) for cultured cells and four volumes for plasma added per sample (500 𝑝g deuterated internal standards per sample). Flasks were then scraped and transferred together with the media plus the methanol and internal standards and subsequently stored at −20°C for 45min to precipitate protein. Subsequently, tubes were centrifuged 2,000 x g for 15min at 4ºC to pellet debris and protein. Lipid mediators were thereafter extracted using solid-phase extraction using an Eclipse Plus C18 column (100 × 4.6 mm × 1.8 μm; Agilent Technologies). [65] Extracted samples were analyzed by LC-MS/MS. An Eclipse Plus C18 was used with a gradient of methanol/water/acetic acid of 55:45:0.01 (vol/vol/vol) that was ramped to 85:15:0.01 (vol/vol/vol) over 10 min and then to 98:2:0.01 (vol/vol/vol) for the next 8 min. This was subsequently maintained at 98:2:0.01 (vol/vol/vol) for 2 min. The flow rate was maintained at 0.4 ml/min. To monitor and quantify the levels of lipid mediators, a multiple reaction monitoring (MRM) method (Supplementary Table 2) was developed with signature ion fragments (m/z) for each molecule monitoring the parent ion (Q1) and a characteristic daughter ion (Q3) followed by an enhanced product ion (EPI) scan for individual lipid spectra identification. [65] Identification was conducted using published criteria, matching retention time to synthetic or authentic standards, MRM peaks with  more than five data points, a signal-to-noise ratio > 3 ([UK/MAP](https://www.nature.com/articles/s41596-022-00801-8#Tab1)). In a subset of samples, the identity of these mediators was further confirmed by matching a minimum of six diagnostic ions in total ion count EPI spectrum to that of a reference standard (Supplementary Figure 2). [41]

### Western Blot

Cell lysates were obtained by growing PS1 cells on 6-well plates either treated with 1µM ATRA or vehicle control (1µM ethanol) in DMEM/F12 supplemented with 10% FBS for 7 days. On the 8^th^ day, cells were washed with cold phosphate buffered saline (PBS) and lysed by adding radio-immunoprecipitation assay (RIPA) buffer with protease inhibitor cocktail (539131, Merck Millipore) and protein extracted by scraping and centrifugation of cell debris. Protein concentration was then determined by standard Bradford protein assay (Bio-rad, USA). Electrophoretic migration was performed on polyacrylamide gels (10% NuPage Bis-Tris gels), followed by wet transfer to a nitrocellulose membrane, washing and saturation (0.05% Tris-buffered saline (TBS, 20-7301-10, Severn Biotech), 5% Tween-20, 5% bovine serum albumin (BSA - A9647, Sigma)) of the membrane, and then hybridization with primary antibody overnight at 4ºC. Thereafter, membranes were washed three times with TBS- 0.05% tween and subsequently incubated with secondary antibody at room temperature for 1h. Before revealing antibody binding by enhanced chemiluminescence (ECL – WBLUF0100, Merck Millipore, USA), the membrane was washed three times with 0.05% TBS-tween. Membranes were then visualized and imaged in an Amersham Imager 600UV (Cytiva Life Sciences). Band densitometry quantification was performed using FIJI Is Just Image J software (FIJI, USA).

###

### Immunocytochemistry (ICC)

Cell slides were obtained by culturing PS1 [2], human cancer associated fibroblasts (CAFs, one patient per subtype) [11], MIAPaCa-2 (CRL-1420, ATCC) or AsPC-1 (CRL-1682, ATCC) cells in 12- or 6-well cell culture plates containing 13mm or 10mm coverslips in the well bottom. All cell lines were STR-profiled and confirmed and tested negative for mycoplasma contamination. After culture and/or treatment, coverslips were recovered, washed in 1X PBS, fixed in 10% Saline Buffered Formalin for 10min, washed 3X with PBS and stored at 4ºC in PBS with 0.01% sodium azide (S2002, Sigma). On the day of staining, cells were permeabilized with ice-cold 100% methanol for 10min at -20ºC and rinsed in PBS. Cells were blocked in PBS 5% goat serum (G9023, Sigma) (secondary antibody species), 0.3% triton X-100 (X100, Sigma) for 1h at room temperature in order to incubate with primary antibodies (Supplementary Table 3) diluted in PBS 1X 1% BSA 0.3% triton X-100 and left overnight at 4ºC. Coverslips were then washed three times with PBS and incubated at room temperature for 1h with respective secondary antibodies. Thereafter, coverslips were washed 3X with PBS and mounted in slides using ProLong Gold anti-fade mountant with 4′,6-diamidino-2-phenylindole (DAPI, P36935, Invitrogen), left overnight at room temperature to dry and imaged using Leica LSM880 confocal microscope. Laser power and background was calculated using negative IgG controls.

### Quantification

For PSC characterization, quantification of intensity was achieved using FIJI Is Just Image J software (FIJI, USA (Schindelin et al., 2012; Schneider et al., 2012)), where average intensity per region of interest (ROI) per cell was calculated. This is to account for considerable cell size change after treatment with ATRA. Hence intensity is normalized by area. A minimum of 4-6 cells per field was quantified and a minimum of 3 fields per condition and biological repeat was collected and averaged for final result.

*Cell Profiler Subcellular Intensity Measuring*

CellProfiler (an open source software) was used to analyze immunocytochemistry images as previously described by McQuin et al. The pipeline runs as follows:

1. A median filter was applied to each individual channel to reduce salt-and-pepper noise while preserving borders as later clear borders will help identify edges for cell and nucleus identification (Supplementary Figure 13B).

2. A reduction of noise was performed in the DAPI channel, which performs non-local means noise reduction. Instead of only using a neighborhood of pixels around a central pixel for denoising, such as in Gaussian filter, multiple neighborhoods are pooled together. The neighborhood pool is determined by scanning the image for regions similar to the area around the central pixel using a correlation metric and a cut-off value. This helped to smooth DAPI-stained nuclei for more precise border detection (Supplementary Figure 13B).

3. Then an identification of primary object was performed using the DAPI channel to create nuclei masks (Supplementary Figure 13C).

4. From the nuclei masks, an expansion of 1µm was performed on the edge of the nucleus. Then a shrinkage of the nucleus mask of 1µm was carried on. Both created two separate masks (Supplementary Figure 13C).

5. A secondary object was created using ⍺-SMA staining at 488nm as a cell shape marker. The software identifies the cells by using the nuclei as a “seed” region, then growing outwards until stopped by the image threshold or by a neighbor. A global minimum cross entropy algorithm thresholding strategy was used as a propagation method to delineate the boundary between neighboring cells (Supplementary Figure 13C).

6. Tertiary objects (masks) were created by defining regions: cytoplasm, nuclear envelope and nucleus. Then pixel intensity within the regions was measured along with other pattern measurements in the 594nm channel for the protein of interest (Supplementary Figure 13D).

### Real Time Quantitative Polymerase Chain Reaction (RT-qPCR) for gene expression analysis

In order to confirm if PS1 cells and CAFs (one patient per subtype) where expressing the enzymes needed for SPM production (ALOX5, ALOX12, ALOX15-1, ALOX15-2, PTGS-1/COX-1 and PTGS-2/COX-2) and their receptors (GPR101, LGR6, ALXR/FPR2, GPR32/DRV1, GPR18/DRV2, GPR37, Chem23/ERV1 and BLT-1/LTB4R), RNA was extracted from these cells after 7 days of treatment with ATRA or vehicle using the Quick-RNA miniprep kit (R1054, Zymo Research) and stored at -80ºC. RNA was then converted to cDNA using random hexamers and oligo(dT) at a 1:1 ratio as enzyme primers, and SuperScript III Reverse Transcriptase (12574026, Invitrogen) and stored at -20ºC. Then, RT-qPCR was performed using QuantiTect pre-designed primers (Qiagen, CA, USA) for the above-mentioned enzymes and receptors. All results are calculated using the ΔΔC_T_ method for normalized relative expression.

### Cell viability MTS Assays

Cells were seeded into the middle 60 wells of a 96 well plate at an optimal density of 2 x 10^3^ and 1.5 x 10^3^ cells per well for cancer and stellate cell lines, respectively. 200 μl of PBS was added to the outside wells to prevent dehydration within the plate during incubation. The cells were seeded in relevant medium at a volume of 200 μl per well and incubated at 37ºC and 5% CO_2_ for 24 hours. Relevant treatments were prepared in cell culture media to treat the cell cultures after 24 hours. Media was then removed from the cultures by tilting the plate and carefully pipetting out the media. Treatments in culture media was then added every 24h and for 72h at 37ºC 5% CO_2_. There were four repeats of each drug on each 96 well plate. To analyze the level of cell viability for each condition, 20 μl of MTS reagent (3-(4,5- dimethylthiazol-2-yl)-5-(3-carboxymethoxyphenyl)-2-(4-sulfophenyl)-2H-tetrazolium) (G3581, Promega) was added. This was left at 37ºC for one hour. MTS is a tetrazolium dye that is metabolized by the mitochondria in viable cells into a brown formazan product. After incubation, the intensity of the color in each well was measured by the absorbance at 492 nm, using a 96-well microplate reader (Infinite® F50, Magellan software). These values were adjusted to allow for the background absorbance and normalized to the control wells, providing a Cell Viability Index relative to the negative control.

### ALOX15 shRNA knockdown Cell Line Generation

Four different (anneal to different human ALOX15 transcripts, termed A-D) ALOX15 human short hairpin RNA (shRNA) green fluorescent protein (GFP) lentiviral plasmids (TL314822, Origene) were used to knock down ALOX15 in PS1 cells. The plasmids are constructed in pGFP-C-shLenti vectors, with chloramphenicol as *E.coli* selection and puromycin as mammalian cell selection antibiotic. shRNA GFP-lentiviral vectors were first transformed into bacteria for cloning, then packaged into virus, transfected into PS1 cells were selected using flow cytometry assisted cell sorting (FACS) for top 10% GFP positive cells.

###

### Bacterial Transformation

One tube with chemically competent One Shot Stb13 *E. coli* (C7373-03, ThermoFisher) was carefully thawed on ice and subsequently mixed with 1µl (100ng) of the OriGENE ALOX15 Human shRNA GFP lentiviral plasmids. One plasmid (A-D) was added per *E. coli* tube and placed back on ice immediately and incubated for 30min. Cells were then heat-shocked at 42ºC for 30-45s in a water-bath and placed back on ice for 2min. 250µl of room temperature (pre-warmed) SOC medium (from *E. coli* kit) was then added to the tubes and incubated at 37ºC for 3h horizontally at 225rpm shaking. From that, 50µl was transferred (both neat and at 1:10 dilution) to Luria broth (LB) agar plates with 30µl/ml chloramphenicol and incubated upside down at 37ºC overnight. One colony from each sample was then transferred to 5ml LB broth with 30µl/ml chloramphenicol in round bottom 14ml polypropylene tubes and incubated overnight at 37ºC with shaking. From that, 500µl from each growth tube was transferred to 250ml of LB broth with 30µl/ml chloramphenicol and incubated overnight at 37ºC with shaking. Plasmid DNA was subsequently extracted using Qiagen Endofree Plasmid Maxi Kit (12362, Qiagen) and DNA quantified using a NanoDrop One (Thermofisher, USA).

###

### Lentiviral Production and Infection

HEK293T cells were seeded into60mm dishes (one per A-D shRNA vectors and one for negative control) to reach 90% confluency after 24h. The following day, 5µg of each shRNA plasmid (A-D), 1.75µg pMD2.G (viral packaging 1 plasmid pCFJ1259 - 1259, Addgene) and 3.25µg CMVR8.74 (viral packaging 2 plasmid pCMV delta R8.2 - 12263, Addgene) was prepared on 470µl optiMEM (31985070, Gibco) with 30µl FuGENE transfection reagent (E231, Promega). This mixture was incubated at room temperature for 10min and then added to the HEK293T cells in 5ml of fresh supplemented medium. After 24h, medium was replaced with fresh supplemented medium. The viral supernatant was collected 24h later, pelleted for cell debris and stored at -80ºC.

###

### Transfection

To infect PS1 cells, cells were seeded into a six well plate to reach 30% confluency in 24h. Lentiviral particles were not titrated as efficiency was presumed high due to previous experience with same reagents (Addgene plasmid #12263; <http://n2t.net/addgene:12263>; RRID:Addgene_12263). Thus, 1 ml of lentiviral supernatant was then added to PS1 cells and incubated for 24h. After this, cells were replaced with fresh medium and infection efficiency confirmed by GFP tag under a fluorescent microscope. PS1 were allowed to grow and expanded into T75cm^3^ flasks and further selected for successful transduction by sorting on the expression of GFP using the BD FACS Aria II (BD Biosciences). Knock down was subsequently assessed by RT-qPCR and western blot.

# ACKNOWLEDGEMENTS

We thank Ed Carter, Lucía Rodríguez and Richard P Grose for their invaluable help with spheroid modelling, Abigail Coetzee for organotypic cultures, Francesco Palmas for his expert technical advice with LC-MS/MS and Esteban Gomez for his invaluable help with lipid mediator profiling analysis. We would like to thank members of Kocher and Dalli laboratory for many discussions and suggestions over the past five years to improve the methodologies and analysis in this research project. We thank patients for the kind donation of samples.

# REFERENCES
